# miR-541 is associated with the prognosis of liver cirrhosis and directly targets JAG2 to inhibit the activation of hepatic stellate cells

**DOI:** 10.1186/s12876-024-03174-2

**Published:** 2024-02-23

**Authors:** Jin-Pei Liu, Shao-Hua Song, Pei-Mei Shi, Xiao-Yu Qin, Bai-Nan Zheng, Shu-Qing Liu, Chen-Hong Ding, Xin Zhang, Wei-Fen Xie, Yi-Hai Shi, Wen-Ping Xu

**Affiliations:** 1 Department of Gastroenterology, Gongli Hospital of Shanghai Pudong New Area, 219 Miaopu Road, 200135 Shanghai, China; 2 Department of General Surgery, Ruijin Hospital, Shanghai Jiaotong University School of Medicine, 200025 Shanghai, China; 3 Department of Gastroenterology, Changzheng Hospital, Naval Medical University, 415 Fengyang Road, 200003 Shanghai, China

**Keywords:** Liver cirrhosis, Prognosis, miR-541, JAG2, Notch signaling pathway

## Abstract

**Background:**

The activation of hepatic stellate cells (HSCs) has been emphasized as a leading event of the pathogenesis of liver cirrhosis, while the exact mechanism of its activation is largely unknown. Furthermore, the novel non-invasive predictors of prognosis in cirrhotic patients warrant more exploration. miR-541 has been identified as a tumor suppressor in hepatocellular carcinoma and a regulator of fibrotic disease, such as lung fibrosis and renal fibrosis. However, its role in liver cirrhosis has not been reported.

**Methods:**

Real-time PCR was used to detect miR-541 expression in the liver tissues and sera of liver cirrhosis patients and in the human LX-2. Gain- and loss-of-function assays were performed to evaluate the effects of miR-541 on the activation of LX-2. Bioinformatics analysis and a luciferase reporter assay were conducted to investigate the target gene of miR-541.

**Results:**

miR-541 was downregulated in the tissues and sera of patients with liver cirrhosis, which was exacerbated by deteriorating disease severity. Importantly, the lower expression of miR-541 was associated with more episodes of complications including ascites and hepatic encephalopathy, a shorter overall lifespan, and decompensation-free survival. Moreover, multivariate Cox’s regression analysis verified lower serum miR-541 as an independent risk factor for liver-related death in cirrhotic patients (HR = 0.394; 95% CI: 0.164–0.947; *P = 0.037).* miR-541 was also decreased in LX-2 cells activated by TGF-β and the overexpression of miR-541 inhibited the proliferation, activation and hydroxyproline secretion of LX-2 cells. JAG2 is an important ligand of Notch signaling and was identified as a direct target gene of miR-541. The expression of JAG2 was upregulated in the liver tissues of cirrhotic patients and was inversely correlated with miR-541 levels. A rescue assay further confirmed that JAG2 was involved in the function of miR-541 when regulating LX-2 activation and Notch signaling.

**Conclusions:**

Dysregulation of miR-541/JAG2 axis might be a as a new mechanism of liver fibrosis, and miR-541 could serve as a novel non-invasive biomarker and therapeutic targets for liver cirrhosis.

**Supplementary Information:**

The online version contains supplementary material available at 10.1186/s12876-024-03174-2.

## Background

Liver cirrhosis is a major global public health problem and an important cause of morbidity and mortality [1]. Liver cirrhosis develops after a long period of inflammation that can be caused by various etiologies, such as hepatitis B or C infections, high alcohol consumption, non-alcoholic fatty liver disease, or autoimmune diseases [1]. The clinical course of cirrhosis is typically characterized by an asymptomatic phase (compensated cirrhosis) and a symptomatic phase (decompensated cirrhosis), which presents with complications such as variceal bleeding, ascites, and hepatic encephalopathy (HE) [2]. The transition from the compensated to the decompensated stage occurs at a rate of 5–7% annually and decreases patients’ life expectancy [2–4]. Therefore, identifying patients with a high risk of decompensation and liver-related death is important for preventing complications and improving prognosis.

Pathologically, liver cirrhosis results from hepatic fibrosis, which is a pathological repair response to chronic injury in various chronic liver diseases [5, 6]. The activation of hepatic stellate cells (HSCs) serves as a pivotal event during hepatic fibrosis [5, 6]. When activated, HSCs acquire pro-fibrogenic and pro-inflammatory properties [7], a process that is modulated by several fibrosis-associated signaling pathways such as transforming growth factor-β (TGF-β)/Smad, Wnt/β-catenin, Hedgehog, and Notch in the injured liver [8]. The dysregulation of these signaling pathways activates HSCs, triggering hepatic fibrosis and eventually the development of liver cirrhosis [8]. Although accumulating evidence has demonstrated the mechanism of the dysregulation of this signaling, additional research is required.

microRNAs (miRNAs) are important regulators of liver physiological processes that negatively regulate target mRNA expression [9]. In chronic liver diseases, miRNAs can regulate fibrogenic signaling pathways in HSCs [8]. In addition, miRNAs are present and stable in serum samples during long-term storage, making them suitable biomarkers for the diagnosis and prognosis assessment of many diseases, including liver cirrhosis [10, 11]. Ren et al. [12] reported that serum miR-744 was inversely correlated with the severity of liver cirrhosis and inhibited liver cirrhosis by targeting TGF-β1. Ma et al. [13] identified miR-98-5p as a novel biomarker suppressing liver fibrosis by targeting TGF-β receptor 1. Serum miR-122 has been reported to predict overall survival in patients with liver cirrhosis [14] and miRNA-21 predicted the transplant-free survival of liver cirrhosis patients [15]. These findings indicate that miRNA can serve as a therapeutic target and disease biomarker for liver cirrhosis, however, there are many unanswered questions that warrant further research.

miR-541 is located at the imprinted delta-like 1 homolog (DLK1)-iodothyronine deiodinase 3 (DIO3) region on human chromosome 14q32, which contains one of the largest miRNA clusters [16–18]. Our previous studies have demonstrated that miR-541 is downregulated in human hepatocellular carcinoma (HCC) tissues and is associated with malignant clinicopathologic phenotypes, recurrence, and survival of patients with HCC [17, 18]. Moreover, it potentiates the response of human HCC to sorafenib treatment by inhibiting autophagy [18]. These findings suggest that the dysregulation of miR-541 plays an important role in HCC. It has also been reported that miR-541 inhibits lung fibrosis and renal fibrosis by targeting different target mRNAs [19–21], indicating that miR-541 is a potential fibrosis-associated miRNA. However, the link between miR-541 and liver fibrosis has not yet been elucidated. In this study, miR-541 was found to be associated with the prognosis of liver cirrhosis and directly targets JAG2 to inhibit the activation of the Notch signaling pathway and HSCs, suggesting that this miRNA could serve as a novel non-invasive biomarker for prognosis prediction and therapeutic target for liver cirrhosis.

## Methods

### Participants and sample collection

There were two sample cohorts in this study. The first cohort included 21 normal liver tissues collected from the marginal donor liver and 20 cirrhotic liver tissues derived from explanted diseased livers at the time of transplantation. These samples were collected from Changzheng Hospital, which is affiliated with Naval Medical University. In the second cohort, 84 patients were hospitalized for liver cirrhosis between April 2014 and February 2022 at Changzheng Hospital. Fifty sex- and age-matched healthy individuals that were evaluated during routine laboratory tests served as a control group. This sample set included sera from 50 healthy controls, 34 patients with compensated cirrhosis, and 50 patients with decompensated cirrhosis. Informed consent was obtained from each participant. To maintain the anonymity of the subjects, the subjects were identified in case report forms by numbers, not by their names. We maintain an identification code sheet with the subject’s code and name. These documents identifying subjects are kept strictly confidential. The study protocol conformed to the ethical guidelines of the 1975 Helsinki Declaration and was approved by the institutional review boards of Naval Medical University (Approval number: 2023SL001).

### The clinical assessment of patients and follow-up

During follow-up, clinical events were recorded, including the development of complications and liver-related death. Clinical decompensation was defined as the occurrence of ascites, spontaneous bacterial peritonitis (SBP), esophageal and gastric varices bleeding, HE, and hepatorenal syndrome (HRS). Survival without liver-related death was defined as the duration from the date of inclusion to liver-related death, with censoring at the time of the last follow-up. The follow-up period ended on 31 January 2022. The serum samples were collected on the hospitalization day of each patient. Biochemical tests, including serum alanine aminotransferase (ALT), aspartate aminotransferase (AST), alkaline phosphatase (ALP), γ-glutamyltransferase (γ-GT), albumin, total bilirubin (TB), creatinine, platelet (PLT) count, prothrombin time (PT) and international normalized ratio (INR) were measured by standard clinical methods. Child-Pugh classification scores was used to assess the severity of the hepatic disease. The baseline characteristics of the 84 included patients and 80 patients with follow-up data are summarized in Supplementary Table 1.

### Cell cultures

LX-2 cells were purchased from Sigma-Aldrich (SCC064) and cultured in Eagle’s minimum essential medium supplemented with 2% FBS. HEK293 cells were maintained in Dulbecco’s modified Eagle’s medium supplemented with 10% fetal bovine serum (FBS).

### Reagents

Small interfering RNAs (siRNAs), miRNA mimics and their double-stranded negative controls (NC), and miRNA inhibitors and their single-stranded scrambled controls (NC inhibitor) were purchased from GenePharma (Shanghai GenePharma Co., Ltd., Shanghai, China).

### Transfection

All of the plasmids, siRNA, miRNA mimic, miRNA inhibitor and their controls were transfected into cells using Lipofectamine2000 (Invitrogen) according to the instruction of manufacturer.

### RNA extraction and real-time PCR

To detect the expression of miR-541 and other genes in cells or tissues, total RNA was isolated using Trizol (Takara, Dalian, China). RNA integrity and purity were determined as the 260/280 and 260/230 nm absorbance ratios and the 28 S/18S rRNA ratio. cDNA was synthesized using the miRNA 1st strand cDNA Synthesis Kit (AG, UK). The expression of various mRNAs was detected using SYBR Green-based real-time PCR (Takara, Dalian, China) and the ABI StepOne Real-time PCR Detection System (Life Technologies). Gene expression was analyzed using the 2^−ΔΔCT^ method. U6 was used as an internal control in LX-2 cells and tissues. For detection of the expression of miR-541 in the serum, total RNA from 200 μL of patient serum samples was extracted using a miRNeasy Serum/Plasma Advanced Kit (Qiagen, Germany). Norgen’s microRNA (cel-miR-39) Spike-In Kit (NORGEN, 59,000) was used to provide a quantified synthetic RNA (cel-miR-39) for spike-in during RNA extraction procedures and subsequent normalization in RT-qPCR assays. After reverse transcription of the sample RNA (with spike-in), the level of cel-miR-39 could be determined by subjecting the cDNA generated to qPCR using SYBR Green. The level of expression of serum miR-541 was normalized to the cel-miR-39 transcript level using the 2^−ΔΔCT^ method. Primer sequences are listed in Supplementary Table 2.

### Western blot

Proteins were extracted using lysis buffer supplemented with protease inhibitor (Roche), separated using sodium dodecylsulfate polyacrylamide gel electrophoresis (SDS-PAGE), and then transferred onto a PVDF membrane (HAHY00010, Millipore) in constant current mode. The membrane was blocked in PBST containing 5% milk for 1 h and then was incubated with the following primary antibody at 4 °C overnight. After that, 1 h incubation with a secondary antibody, signals were quantitated using an Odyssey infrared imaging system (LI-COR) at 700 or 800 nm.

### Cell proliferation assay

Cell Counting Kit-8 (Dojinodo, Shanghai, China) was used to analyze the proliferation of LX-2 cells.

### Hydroxyproline (Hyp) level detection

Hyp in the culture supernatant of LX-2 receiving different treatment was detected utilizing a commercial Hyp assay kit (Biyotime, Suzhou, China) according to the instruction of manufacturer.

### Construction of the reporter and luciferase assay

The full-length of 3′UTR of human JAG2 were subcloned into the pmirGLO vector to construct the luciferase reporter plasmid harboring the JAG2 3’UTR. The miR-541 binding site in the JAG2 3′UTR reporter vector was mutated, synthesized and subcloned into the pmirGLO vector. The luciferase activities were measured by the Dual-Luciferase Reporter Assay (Promega, Madison, WI, USA).

### Statistical analysis

Continuous variables were expressed as median with range. Comparisons of data with normal distribution between two groups were performed using Student’s *t*-tests. Mann-Whitney U test was used to compare two groups with variables being not normally distributed. Comparisons among three groups were made by Kruskal-Wallis non-parametric test and the following pairwise comparisons were achieved by Nemenyi test. A Kaplan-Meier analysis and Log-rank test were performed to compare the survival probabilities between different groups. A univariate Cox’s regression analysis was first used to identify the prognostic factors for cirrhotic patients. Those significant factors (*P* < 0.05) were then analyzed using a multivariate Cox’s regression analysis. Results of Cox regression are presented as adjusted hazards ratios (HR) with 95% CI and two-sided *P*-values. Spearman’s correlation coefficients were calculated between numerical variables. Statistical tests were two-tailed and a *P*-value less than 0.05 was considered statistically significant. All analyses were performed with SPSS version 22.0 (SPSS, Chicago, IL, USA).

Detailed descriptions of the materials and methods are included in the online supplementary materials and methods.

## Results

### miR-541 is downregulated in the liver tissues and sera of patients with liver cirrhosis and is associated with liver function and the prognosis of patients

**Fig. 1:**
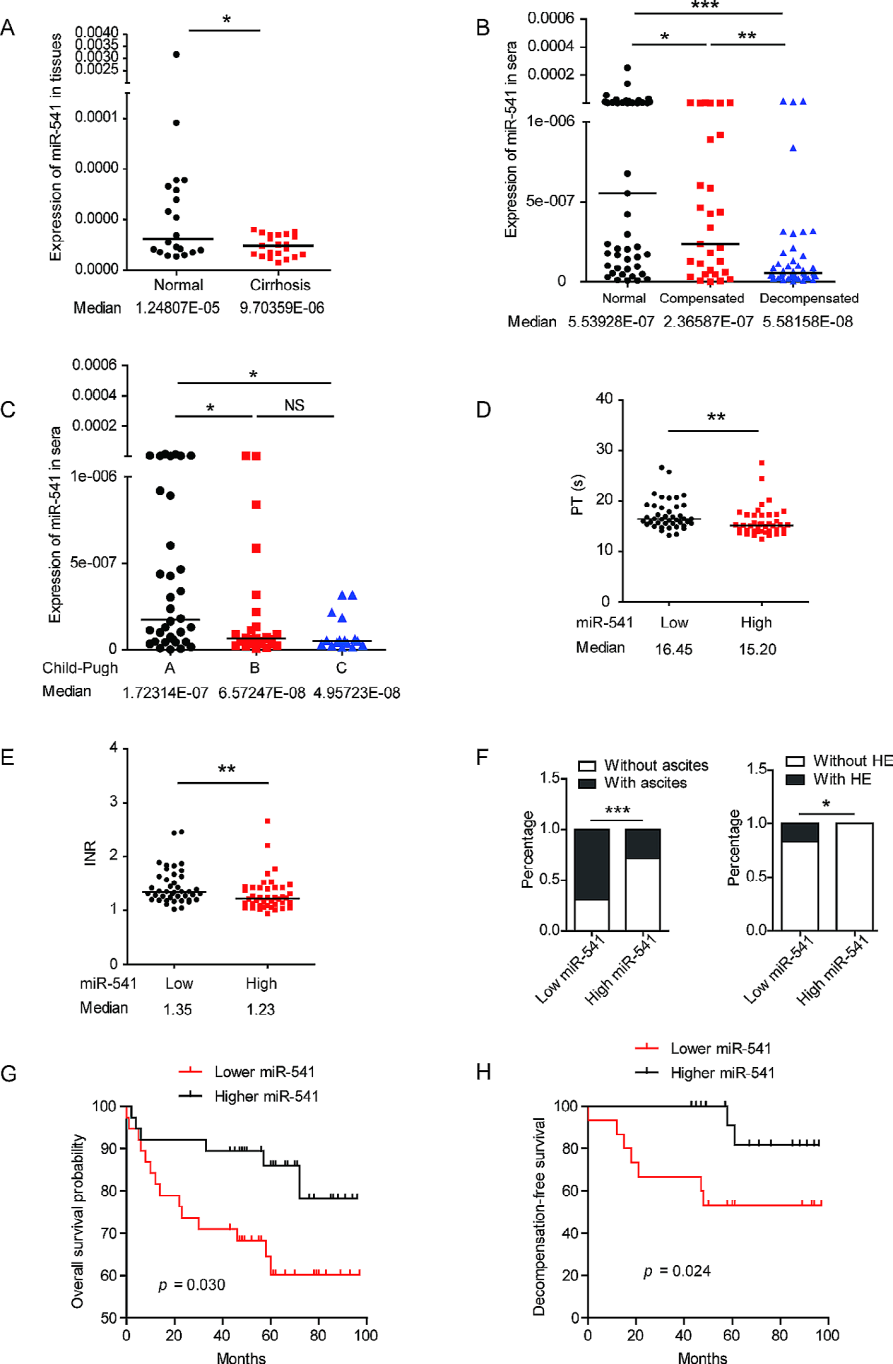
miR-541 is downregulated in the liver tissues and sera of patients with liver cirrhosis and is associated with the liver function and prognosis of patients. (**A**) Real-time PCR analysis of miR-541 expression in the cirrhotic liver tissues and normal liver tissues, with medians of 1.24807 × 10^− 5^ and 9.70359 × 10^− 6^, respectively; *p* = 0.0406, non-parametric Mann-Whitney test. (**B**) The expression of miR-541 in the sera of healthy controls, patients with compensated cirrhosis, and patients with decompensated cirrhosis. Comparisons among the three groups were made by Kruskal-Wallis non-parametric test and the following pairwise comparisons were achieved by Nemenyi test. * *p* < 0.05, ** *p* < 0.05, *** *p* < 0.001. (**C**) miR-541 levels were downregulated with the aggravation of the Child-Pugh classification. Comparisons among the three groups were made by Kruskal-Wallis non-parametric test and the following pairwise comparisons were achieved by Nemenyi test. * *p* < 0.05, NS, not significant. (**D** and **E**) Statistical analysis of PT (**D**) and INR (**E**) values in patients with high and low miR-541 expression. The cut-off value to separate patients with high and low miR-541 expression was the median value in 84 cirrhotic patients. ** *p* < 0.01, non-parametric Mann-Whitney test. (**F**) Percentage of patients with or without ascites (left) and HE (right) at enrollment in the subgroup of patients with low and high miR-541 levels. * *p* < 0.05, *** *p* < 0.001 by Fisher test. (**G** and **H**) Overall survival (OS) (**G**) and decompensated-free survival (**H**) were compared between cirrhotic patients with low and high miR-541 expression using the log-rank test. The median value was used as a cut-off to separate patients with high and low miR-541 expression

In cohort 1 with normal liver tissues (*n* = 21) and cirrhotic liver tissues (*n* = 20), the expression of miR-541 was significantly decreased in the cirrhotic liver tissues compared with normal liver tissues (Fig. 1A). In cohort 2 with sera from 50 healthy controls, 34 patients with compensated cirrhosis, and 50 patients with decompensated cirrhosis, the expression of miR-541 was significantly decreased in the cirrhotic patients compared with the healthy controls (Fig. S1). Moreover, serum miR-541 value was significantly decreased in patients with decompensated liver cirrhosis compared to compensated cirrhotic patients (Fig. 1B). Similarly, serum miR-541 levels significantly decreased with the upgrading of Child-Pugh class (Fig. 1C). In addition, high miR-541 levels were associated with better coagulation function, as shown by PT and INR (Fig. 1D, E). The serum miR-541 levels were also negatively correlated with the presence of ascites and HE when patients were included (Fig. 1F). Importantly, the lower expression of serum miR-541 was associated with shorter overall survival and decompensation-free survival (Fig. 1G, H). To further evaluate the independent prognostic value of miR-541 in liver cirrhosis, a univariate Cox’s regression analysis was performed firstly to determine the risk factors for liver-related death in cirrhotic patients, using several variables, including age, gender, etiology, PLT count, INR, PT, ALT, AST, ALP, γ-GT, albumin, TB, and serum miR-541 levels. The following multivariate analysis with the significant factors in univariate analysis, including age, TB, PT, INR, and serum miR-541 levels, indicated older age (HR = 2.873; 95% CI: 1.240–6.657; *P* = 0.014) and lower serum miR-541 (HR = 0.394; 95% CI: 0.164–0.947; *P* = 0.037) as independent risk factors for liver-related death (Table 1). Collectively, these results demonstrated that the downregulation of miR-541 is an important event in the progression of liver cirrhosis.

**Table 1:** Multivariate Cox’s regression analysis of the risk factors associated with the liver-related death in patients with liver cirrhosis

Factor	Category	HR	95% CI	*P* value
Serum miR-541 levels	< median	1	0.164–0.947	0.037
	≥ median	0.394		
Age (years)	< 60	1	1.240–6.657	0.014
	≥ 60	2.873		

HR: hazard ratio; CI: confidence interval

### miR-541 inhibits the proliferation and activation of LX-2 cells

**Fig. 2:**
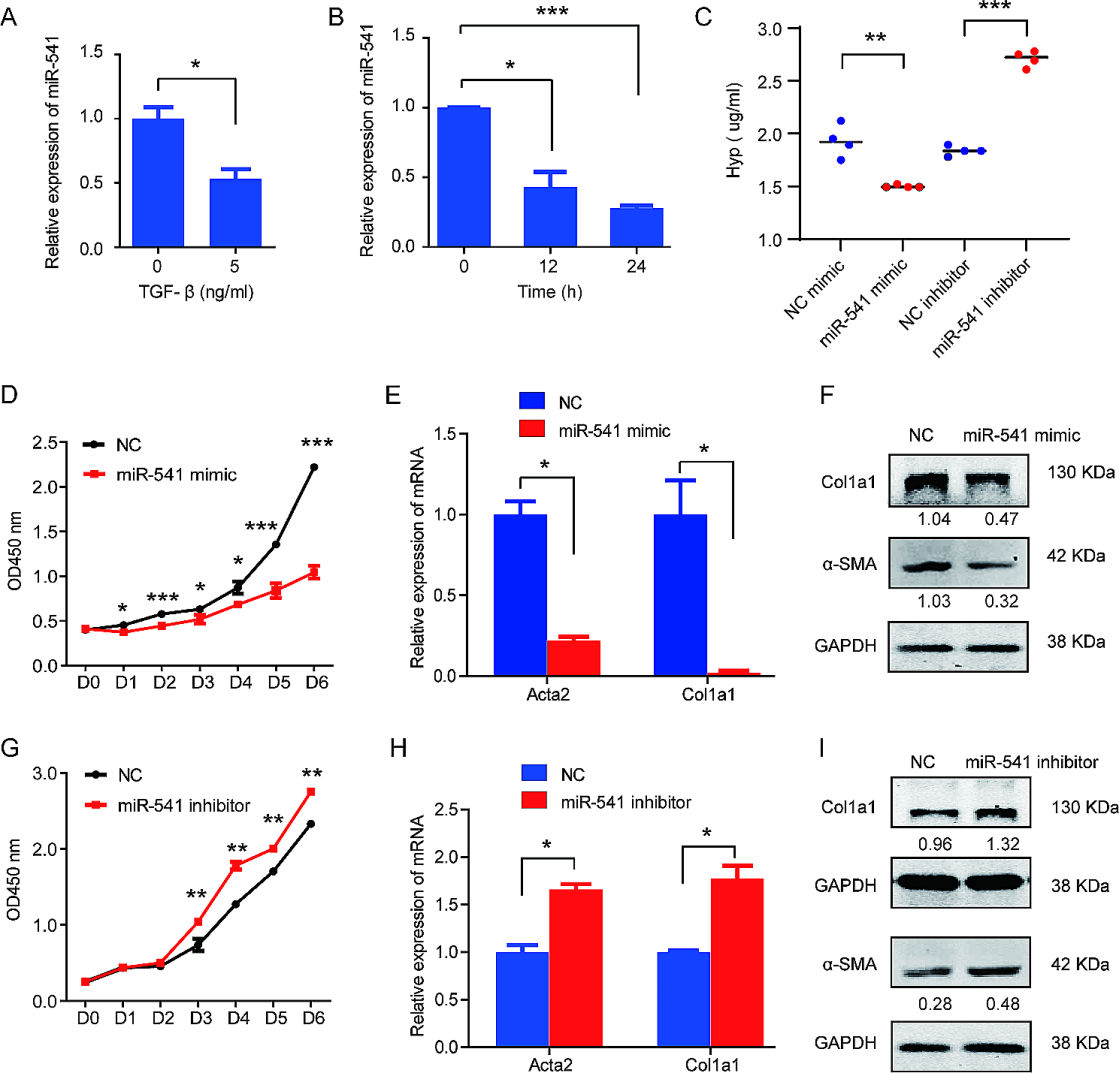
miR-541 inhibits the proliferation and activation of LX-2 cells. (**A**) The expression of miR-541 was detected in LX-2 cells treated with 5 ng/ml TGF-β and the control group (0). (**B**) miR-541 expression was detected in LX-2 cells treated with TGF-β (5 ng/ml) at 0 h, 12 h, and 24 h. (**C**) miR-541 mimic reduced, while miR-541 inhibitor increased the secretion of hydroxyproline in LX-2 cells treated by TGF-β (5 ng/ml). (**D**) Transfection of miR-541 mimic inhibited the proliferation of LX-2 cells treated by TGF-β (5 ng/ml). (**E**) miR-541 mimic reduced the expression of mRNA of Acta2 and Col1a1 in LX-2 cells treated by TGF-β (5 ng/ml). (**F**) Western blot analysis was performed in LX-2 cells treated by TGF-β (5 ng/ml) and transfected with miR-541 mimic or negative control (NC) to detect α-SMA and Col1a1 expression. The bands were quantified by image J software, and the intensity of the bands was calculated as the ratio of the indicated band to the GAPDH band. (**G**) Transfection of miR-541 inhibitor enhanced the proliferation of LX-2 cells treated by TGF-β (5ng/ml). (**H**) miR-541 inhibitor promoted the expression of mRNA of Acta2 and Col1a1 in LX-2 cells treated by TGF-β (5ng/ml). (**I**) miR-541 inhibitor enhanced the protein expression of α-SMA and Col1a1 in LX-2 cells treated by TGF-β (5ng/ml). * *p* < 0.05, ** *p* < 0.01 and *** *p* < 0.001 by two-tailed Student’s t-test. Experiments were performed in triplicate and data are presented as means ± SD.

To investigate the roles of miR-541 in the activation of hepatic stellate cells, the expression of miR-541 was detected in the human HSC line LX-2 at the quiescent status and the activated status treated by TGF-β. The results demonstrated that miR-541 levels were downregulated with the activation of LX-2 in a time-dependent manner (Fig. 2A, B). We then evaluated the effects of miR-541 on the proliferation, activation and collagen synthesis ability of LX-2 by transfecting cells with a miR-541 mimic or miR-541 inhibitor (Fig. S2). Overexpression of miR-541 significantly inhibited the proliferation and hydroxyproline secretion of LX-2, and reduced the mRNA and protein expression of fibrosis markers α-smooth muscle actin (α-SMA) and Collagen type l alpha 1(Col1a1) in LX-2 (Fig. 2C-F). In contrast, the inhibition of miR-541 promoted the proliferation and hydroxyproline secretion, as well as increased the expression of fibrosis-related markers in LX-2 cells (Fig. 2C and G-I). These data suggest that miR-541 functions as a suppressor of HSCs.

### miR-541 suppresses the notch signaling pathway

**Fig. 3:**
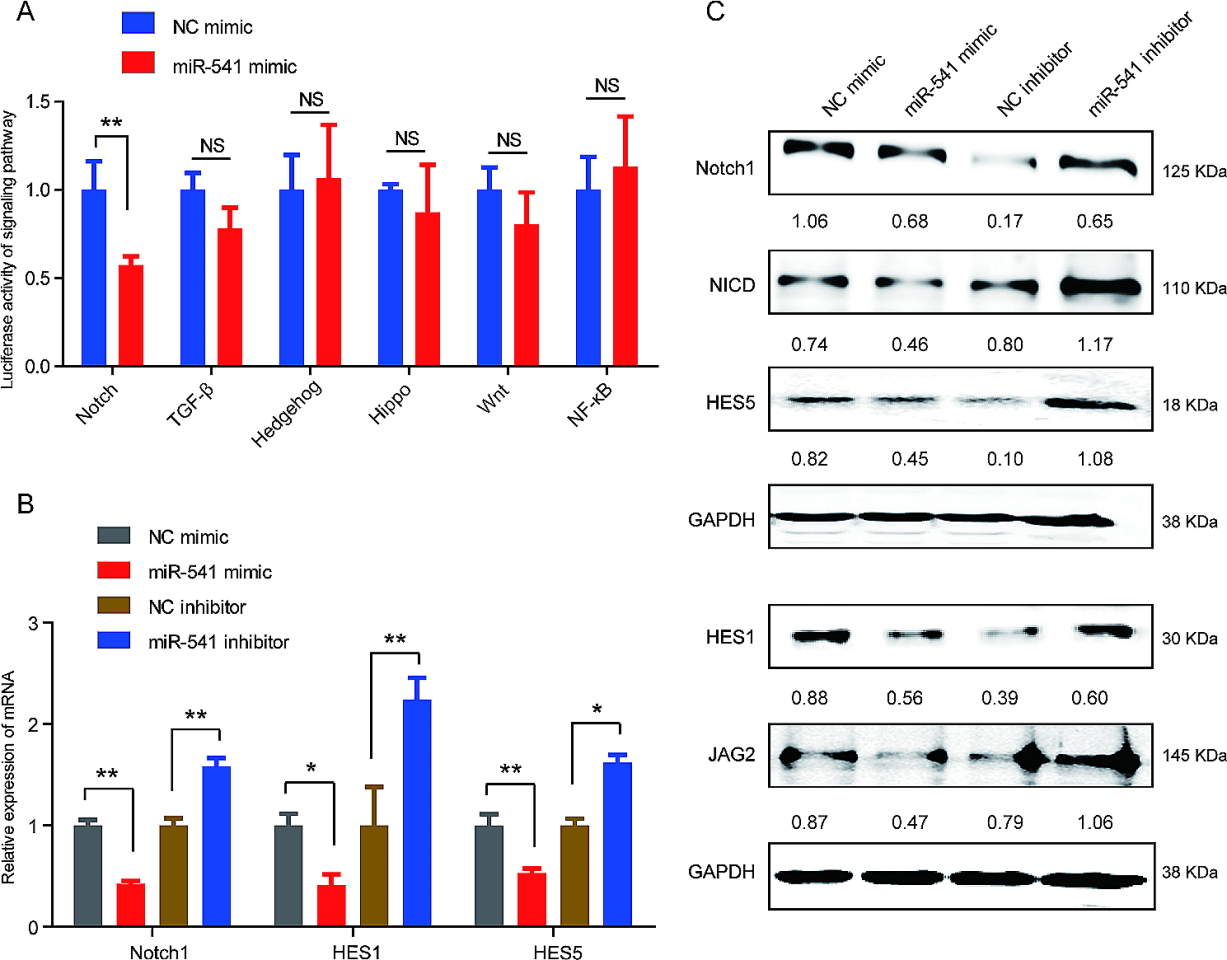
miR-541 suppresses the Notch signaling pathway in HSCs. (**A**) The effects of miR-541 on the luciferase activities of reporters of liver fibrosis-associated signaling pathways including Notch, TGF-β, Hedgehog, Hippo, Wnt, and NF-κB were detected in LX-2 cells. (**B** and **C**) Real-time PCR (**B**) and Western blot analysis (**C**) were performed to detect the expression of key effectors of Notch signaling, including Hes1, Hes5, Notch1, and JAG2 in LX-2 cells transfected with miR-541 mimic, miR-541 inhibitor, and their corresponding negative control. * *P* < 0.05 and ** *P* < 0.01, NS, not significant, two-tailed Student’s *t*-tests. Experiments were performed in triplicate and data are presented as means ± SD.

To investigate the mechanism by which miR-541 inhibited the activation of HSCs, we detected the effects of miR-541 on the luciferase activities of reporters of liver fibrosis-associated signaling pathways, including Notch, TGF-β, Hedgehog, Hippo, Wnt, and NF-κB signaling in LX2 cells. The results showed that miR-541 overexpression significantly inhibited the luciferase activities of reporters of Notch signaling, while reporters of other signaling pathways did not respond to miR-541 overexpression (Fig. 3A). Furthermore, miR-541 overexpression significantly reduced the mRNA and protein expression of key effectors of Notch signaling, including Hes1, Hes5, Notch1 and JAG2, while the inhibition of miR-541 exerted the opposite effects in LX-2 cells (Fig. 3B, C). These data verify that miR-541 serves as a suppressor of the Notch signaling pathway in liver fibrosis.

### JAG2 is a direct target of miR-541

**Fig. 4:**
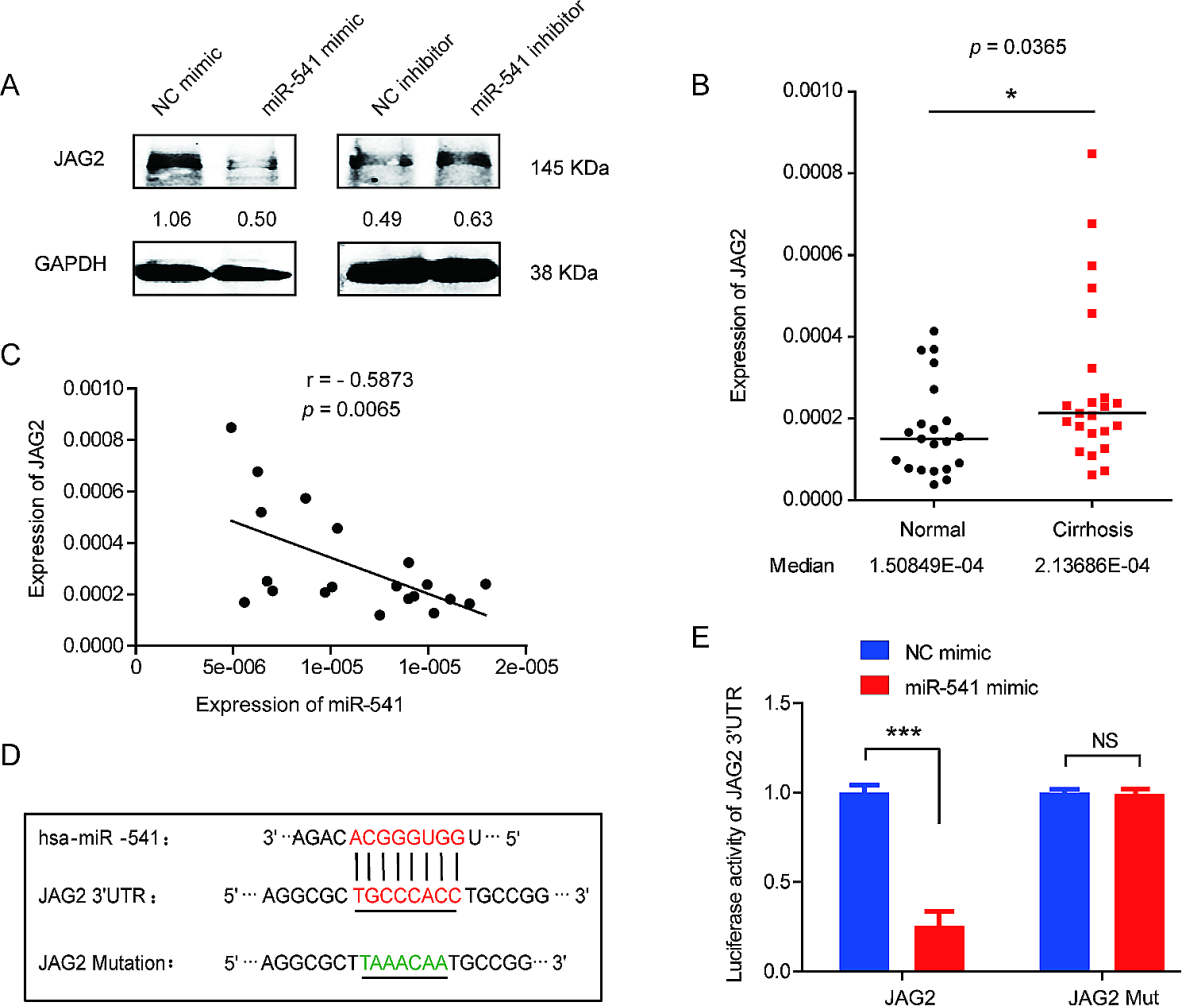
JAG2 is a direct target of miR-541. (**A**) Western blot analysis of JAG2 expression in LX-2 cells treated by TGF-β (5ng/ml) and transfected with the miR-541 mimic or miR-541 inhibitor. (**B**) Real-time PCR analysis of JAG2 expression in the cirrhotic liver tissues and normal liver tissues, with medians of 1.50849 × 10^− 4^ and 2.13686 × 10^− 4^, respectively; *p* = 0.0365, non-parametric Mann-Whitney test. (**C**) The correlation of JAG2 expression and miR-541 levels in the cirrhotic liver tissues. *p* = 0.0065 by Spearman’s correlation coefficient test. (**D**) Predicted sequential pairing of the target region and the parallel mutant sequences in the 3’UTR of JAG2 (bottom) with miR-541 (top) using the prediction programs. Seed sequences are highlighted, and mutant sequences are shown in green. (**E**) Effect of miR-541 on the JAG2 3’UTR. Luciferase reporter plasmids carrying the JAG2 3’UTR (JAG2), and JAG2 3’UTR with miR-541 target-sequence mutation (JAG2 Mut) were co-transfected into HEK293 cells with the miR-541 mimic or NC. *** *p* < 0.001 using two-tailed Student’s *t*-tests. NS, not significant. Experiments were performed in triplicate and data are presented as means ± SD.

We performed a bioinformatics analysis to identify the putative candidate mRNA target of miR-541 that is involved in HSC activation and related to the Notch signaling pathway. All four of the miRNA prediction programs, including Targetscan (http://www.targetscan.org/), microRNA.org (http://www.microrna.org/), ENCORI (starBASE v2.0) (http://starbase.sysu.edu.cn/), and mirWalk (http://mirwalk.umm.uni-heidelberg.de/) found miRNA-target interactions between miR-541 and JAG2 which functions as an important ligand of Notch signaling (Fig. S3). As expected, the level of the JAG2 mRNA and protein decreased in LX-2 cells after the ectopic expression of miR-541 and increased by the miR-541 inhibitor (Fig. S4, 4A). JAG2 expression significantly increased in cirrhotic liver tissues compared with normal liver tissues (Fig. 4B) and was inversely correlated with the miR-541 levels in cirrhotic liver samples (Fig. 4C). Using miRNA prediction programs, we identified a predicted binding site for miR-541 in the 3’ untranslated regions (UTR) of JAG2 mRNA (Fig. 4D). Consistent with our prediction, miR-541 overexpression decreased the luciferase activity of constructs containing the wild-type JAG2 3’ UTR, while point mutation of the target sequences on the 3’ UTRs diminished the effect of miR-541 on luciferase activity in the reporter assay (Fig. 4E). This indicates that JAG2 is a direct target of miR-541 in liver cirrhosis.

### JAG2 promotes the proliferation and activation of LX-2 cells

**Fig. 5:**
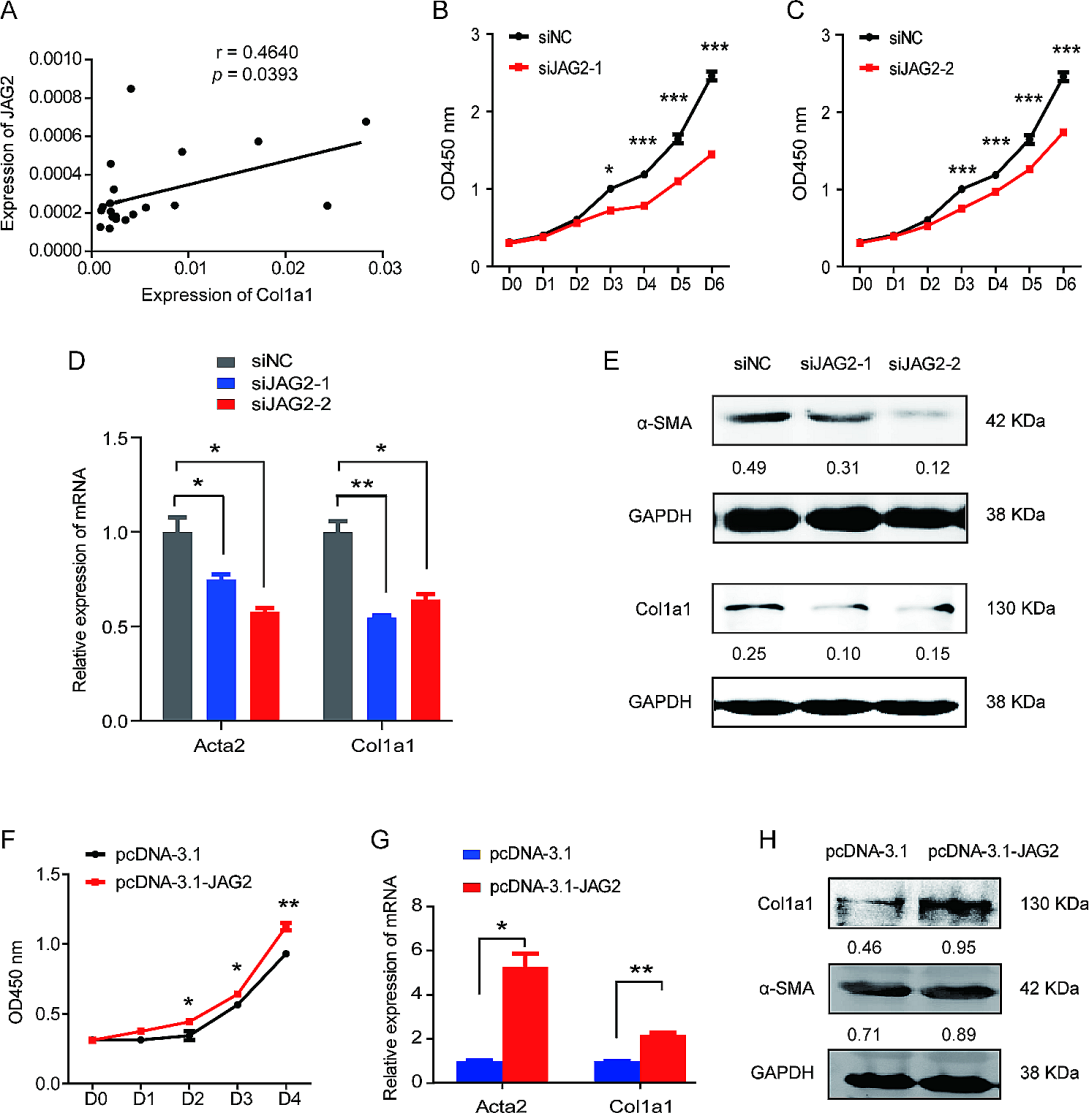
JAG2 promotes the proliferation and activation of LX-2 Cells. (**A**) The correlation of JAG2 expression and fibrosis marker Col1a1 in liver tissues of cirrhotic patients. *p* = 0.0393 by Spearman’s correlation coefficient test. (**B** and **C**) siRNAs interfering JAG2 siJAG2-1 (**B**) and siJAG2-2 (**C**) inhibited the proliferation of LX-2 cells treated by TGF-β (5ng/ml). * *p* < 0.05 and *** *p* < 0.001 by two-tailed Student’s t-test. (**D** and **E**) siJAG2-1 and siJAG2-2 suppressed the mRNA (**D**) and protein (**E**) expression of α-SMA and Col1a1 in LX-2 cells treated by TGF-β (5ng/ml). * *p* < 0.05 and ** *p* < 0.01 by two-tailed Student’s t-test. (**F**) Overexpression of JAG2 promoted the proliferation of LX-2 cells treated by TGF-β (5ng/ml). * *p* < 0.05 and ** *p* < 0.01 by two-tailed Student’s t-test. (**G** and **H**) Overexpressing JAG2 enhanced the mRNA (**G**) and protein (**H**) expression of α-SMA and Col1a1 in LX-2 cells treated by TGF-β (5ng/ml). * *p* < 0.05 and ** *p* < 0.01 by two-tailed Student’s t-test. The above experiments were performed in triplicate and data are presented as means ± SD.

Although the Notch signaling pathway has been reported to play an important role in liver fibrosis, the function of JAG2 on the activation of HSCs has not yet been reported. To confirm that JAG2 was a functional target of miR-541 in liver cirrhosis, we first evaluated the correlation of JAG2 expression and fibrosis marker and found that JAG2 expression was negatively correlated with Col1a levels (Fig. 5A). Additionally, the expression of JAG2 was detected in quiescent and activated LX-2 and the results indicated that JAG2 expression was upregulated with the activation of LX-2 in dose- and time-dependent manners (Fig. S5). We then evaluated the effects of JAG2 on the proliferation and activation of LX-2 by transfecting cells with JAG2 siRNAs and an overexpression plasmid (Fig. S6). As expected, inhibiting JAG2 significantly inhibited the proliferation of LX-2 and increased the mRNA and protein expression of α-SMA and Col1a1 in LX-2 (Fig. 5B-E). In contrast, overexpression of JAG2 promoted the proliferation and activation of LX-2 cells (Fig. 5F-H), as well as the mRNA and protein expression of key effectors of Notch signaling including Hes1, Hes5, and Notch1 in LX-2 cells (Fig. S7). These data identify JAG2 as a functional target of miR-541 in liver cirrhosis.

**Fig. 6:**
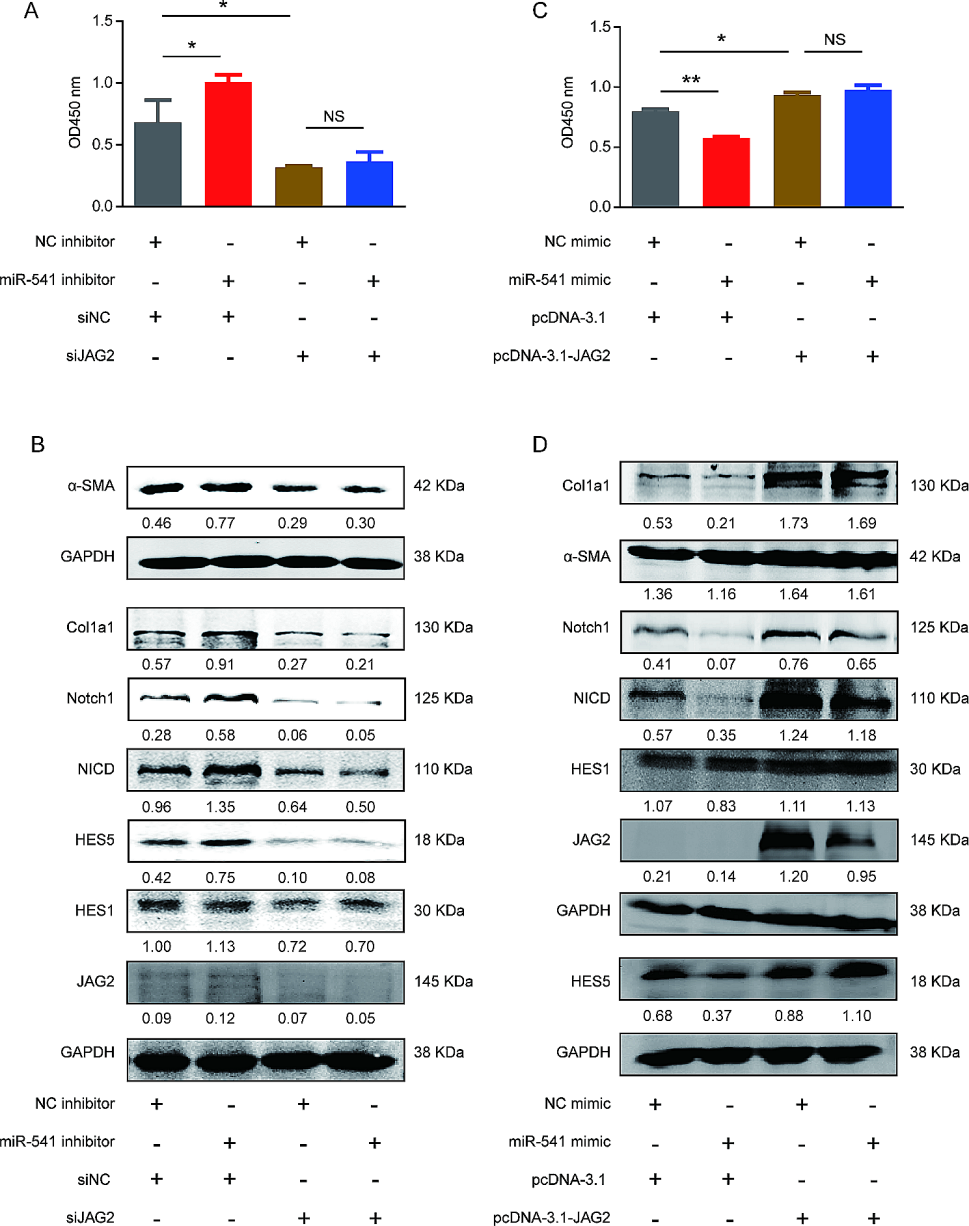
JAG2 partially mediated the effects of miR-541 on the proliferation and activation of HSCs. (**A**) siJAG2 partially reversed the effects of miR-541 inhibition on the proliferation of LX-2 cells. (**B**) The effects of the miR-541 inhibitor on the expression of α-SMA, Col1a1, Notch1, NICD, HES1, and HES5 of LX-2 cells were partially reversed by siJAG2. (**C**) JAG2 overexpression partially reversed the effects of miR-541 promotion on the proliferation of LX-2 cells. (**D**) The effects of miR-541 mimic on the expression of fibrosis markers and the expression of Notch signaling effectors of LX-2 cells were partially reversed by the JAG2 overexpression plasmid. All LX-2 cells were treated with TGF-β (5ng/ml). * *p* < 0.05 and ** *p* < 0.01, two-tailed Student’s *t*-tests. NS, not significant. Experiments were performed in triplicate and data are presented as means ± SD.

### miR-541 inhibits the activation of HSCs by downregulating JAG2

To explore the role of JAG2 in the effects of miR-541 on HSCs, a series of rescue assays were performed by co-transfecting the miR-541 inhibitor and siJAG2 or the miR-541 inhibitor and JAG2 overexpressing plasmid. The effects of the miR-541 inhibitor on increasing the proliferation, the expression of fibrosis markers, and the expression of Notch signaling effectors of LX-2 cells were substantially reduced in cells transfected with siJAG2 (Fig. 6A, B and S8). In contrast, the effects of miR-541 on reducing proliferation, and the expression of fibrosis markers and expression of Notch signaling effectors of LX2 cells were substantially reversed in cells transfected with JAG2 overexpressing plasmid (Fig. 6C, D and S9). Overall, the downregulation of JAG2 at least partially contributes to the functional effects of miR-541 on the activation of HSCs and the Notch signaling pathway.

## Discussion

A tool that can predict the clinical outcomes of liver cirrhosis can help to identify patients with a high risk of clinical decompensation and liver-related morbidity and identify needs related to patient treatment, monitoring, and surveillance [22]. Although Child-Pugh staging has been widely used to evaluate liver reserve function and predict the outcomes of cirrhotic patients, it has some defects and its predictive value must be further identified [23]. An ideal prognostic tool should be reproducible, objective, and readily available [15]. To date, a variety of non-invasive methods have been developed to establish risk scores and predict cirrhosis-related complications and morbidity [24–26]. Stable in the serum, several studies have found that serum miRNAs are associated with liver function and can predict liver-related events [12–15]. This research mostly focuses on predictive tools for liver-related morbidity, and there are limited prognostic tools to predict the onset of decompensation, which substantially reduces the life expectancy of cirrhotic patients [27]. In this study, tissue and serum miR-541 were significantly downregulated in cirrhotic patients compared with healthy controls, and serum miR-541 was further decreased in patients at a more severe disease stage. Its expression was positively associated with liver function and negatively associated with the development of complications. Importantly, the lower expression of serum miR-541 was associated with shorter overall survival and clinical decompensation-free survival, and was identified as the independent risk factor of liver-related death in patients, indicating that miR-541 could be a potential full-featured biomarker for liver function evaluation and prognosis assessment.

Accumulating evidence has demonstrated that miRNAs can regulate fibrogenic signaling pathways in HSCs, thus activating HSCs or maintaining the quiescent phenotype of normal HSCs [8, 11]. Therefore, investigating miRNAs that activate or stabilize HSCs could provide novel targets for hepatic fibrosis treatment. miR-541 is a well-explored miRNA that is involved in various physiological processes [28–30] and serves as a tumor suppressor in many cancers [31], including HCC [18, 32, 33]. Additionally, miR-541 was reported to negatively regulate the signaling pathways involved in HSCs activation, such as the Wnt signaling [29, 31, 32] and TGF-β pathways [21]. Intriguingly, miR-541 could be a fibrosis-related miRNA since it inhibits pulmonary fibrosis and renal fibrosis by targeting the epithelial-mesenchymal transition (EMT) and TGF-β pathways [19–21]. Therefore, we speculated that miR-541 could play a role in suppressing HSCs in liver fibrosis, which has not yet been explored. As expected, miR-541 inhibited the proliferation and activation of HSCs. After screening the well-defined signaling involved in the pathogenesis of liver fibrosis, we clarified that miR-541 suppressed Notch signaling by targeting JAG2. These findings confirm that miR-541 is a novel regulator of HSCs and a potential treatment target for liver fibrosis. Having small size and low molecular weight, miRNAs may be formulated into an effective delivery system easily and become attractive options for treatment of diseases [34]. The recent years have witnessed a great progress in the development of in-vivo delivery system of miRNA, including viral delivery system and non-viral delivery system, such as nanoparticles [34, 35]. It must be taken into account that delivering naked miRNAs may hinder miRNA uptake by cells due to negatively charged groups of miRNAs and lead to undesirable off-target or on-target effects [35]. Fortunately. functionalizing nanoparticles with cell-specific ligands allows delivering miRNAs to specific cells, thus reducing off-target effects [35]. These progresses may pave the way of miR-541 as a treatment target in liver cirrhosis.

JAG2 is a canonical ligand of Notch signaling, which functions as a regulator of physiological processes such as cell differentiation [36, 37] and an oncogene in many tumors such as HCC [38] and pancreatic adenocarcinoma [39]. The deregulation of the Notch cascade plays an important role in liver fibrosis by modulating the activation of HSCs and myofibroblast [40–42], the macrophage polarization [43] and the endothelial function [44]. In addition, the modulation of Notch signaling by miRNA in HSCs activation has recently been reported. Yang et al. [45] found that miR-200a activated HSCs by targeting the SIRT1/Notch1 signal pathway and the loss of miR-200a was correlated with Notch1 down-regulation in HSCs, indicating that deregulation of the miRNA-Notch axis could exert a pivotal function in liver fibrosis. As a key component of the Notch cascade, the role of JAG2 in hepatic fibrosis has not been reported. In this study, we found that JAG2 expression significantly increased in cirrhotic liver tissues and activated HSCs and was positively correlated with Col1a1 in human cirrhotic tissues. Subsequent functional experiments indicated that JAG2 promoted the proliferation and activation of HSCs. Mechanically, JAG2 partially mediated the effects of miR-541 in regulating HSCs activation. These findings first elucidate the role of JAG2 in hepatic fibrosis and provide an unreported target for miR-541 and another novel target for hepatic fibrosis therapy.

There are some limitations of the present study. One of the limitations is its retrospective nature. As a retrospective study, sample size and power calculation as well as blinding and randomization are hard to perform, which may lead to bias. Another limitation is the relatively small sample size in cohort one, due to the difficulty of obtaining the biopsy tissues of cirrhotic liver. Finally, the single center nature limits generalizability, to some extent, to other cirrhotic population. Hence, future prospective trials in larger cohorts in multicenter are needed to further investigate the clinical significance of miR-541 in liver cirrhosis.

## Conclusions

This study highlights the clinical association and biological significance of miR-541 in liver cirrhosis and elucidates a previously unrecognized molecular mechanism underlying hepatic fibrosis. These findings establish miR-541 and JAG2 as two novel therapeutic targets and prognostic markers for liver cirrhosis. As such, approaches designed to manipulate dysregulation of the miR-541-JAG2 axis could be a potential strategy for treating liver cirrhosis.

### Electronic supplementary material

Below is the link to the electronic supplementary material.

Supplementary Material 1

Supplementary Material 2

## Data Availability

The datasets generated during and/or analyzed during the current study are available from the corresponding author on reasonable request.

## Abbreviations

α-SMA: α-smooth muscle actin;
Col1a1: Collagen type l alpha 1
HCC: Hepatocellular carcinoma;
HE: Hepatic encephalopathy;
HR: Hazard ratio
HSCs: Human hepatic stellate cells;
INR: International normalized ratio;
miRNAs: microRNAs;
PT: Prothrombin time;
