# Supplementary material for: miR-541 is associated with the prognosis of liver cirrhosis and directly targets JAG2 to inhibit the activation of hepatic stellate cells
Source: BMC Gastroenterol. 2024 Feb 23;24:84. doi: 10.1186/s12876-024-03174-2 (PMC10893617; doi:10.1186/s12876-024-03174-2)
Supplement: Supplementary file 1 — Supplementary Material 1 [file 12876_2024_3174_MOESM1_ESM.docx]

**Supplementary materials**

**Supplementary methods**

**Reagents**

Small interfering RNAs (siRNAs), miRNA mimics and their double-stranded negative controls (NC), and miRNA inhibitors and their single-stranded scrambled controls (NC inhibitor) were purchased from GenePharma (Shanghai GenePharma Co., Ltd., Shanghai, China). Overexpression plasmid of human JAG2 and controlled plasmid were bought from Youbio Biological Technology Company. All sequences are listed in supplementary Table 3.

**Cell proliferation assay**

Cell Counting Kit-8 (Dojinodo, Shanghai, China) was used to analyze the proliferation of LX-2 cells. Cells were seeded into 96-well plates with 100 mL culture medium. The 10 mL of CCK-8 solution was added to the cells at specific time points and cells were incubated for 1 h at 37℃. The reaction product was quantified according to the manufacturer’s instructions.

**Construction of the reporter and luciferase assay**

The full-length of 3′UTR of human JAG2 were subcloned into the pmirGLO vector to construct the luciferase reporter plasmid harboring the JAG2 3’UTR. The miR-541 binding site in the JAG2 3′UTR reporter vector was mutated, synthesized and subcloned into the pmirGLO vector. Both of the plasmids were bought from Youbio Biological Technology Company.

To investigate the effect of miR-541 on the luciferase reporter plasmid harboring the wild type and mutant JAG2 3’UTR, HEK293 cells cultured in 24-well plate were co-transfected with 20 pmol/well miR-541 mimic or negative control (NC) and 400 ng/well pmirGLO plasmids using 2 μl/well Lipofectamine2000 (Invitrogen). Forty-eight hours after transfection, the Renilla and firefly luciferase activities were measured by the Dual-Luciferase Reporter Assay (Promega, Madison, WI, USA) with a luminometer (Synergy™ 4 Hybrid Microplate Reader, BioTek, USA). The luciferase score was calculated by normalizing the luciferase signal of Renilla against that of firefly. At least three independent experiments were carried out for each condition.

To detect the effect of miR-541 on the Notch, Wnt, Hippo, NF-κB, Hedgehog and TGF-β pathways, the pathway reporter plasmids^1-7^ of these pathways were co-transfected into LX-2 cells with pRL-SV40 using Lipofectamine 2000 (Life Technology). Luciferase activities were measured using Dual-Luciferase Reporter Assay (Promega) 24 hours later with a luminometer (BioTek, VT).The luciferase score was calculated by normalizing the firefly luciferase signal to that of renilla luciferase. At least three independent experiments were carried out for each condition.

**Western blot**

Proteins were extracted using lysis buffer supplemented with protease inhibitor (Roche), separated using sodium dodecylsulfate polyacrylamide gel electrophoresis (SDS-PAGE), and then transferred onto a PVDF membrane (HAHY00010, Millipore) in constant current mode. The membrane was blocked in PBST containing 5% milk for 1 h and then was incubated with the following primary antibody at 4°C overnight. After that, 1 h incubation with a secondary antibody (donkey-anti-mouse or donkey-anti-rabbit, IRDye 700 or IRDye 800, respectively), signals were quantitated using an Odyssey infrared imaging system (LI-COR) at 700 nm or 800 nm. The bands were quantified by image J software, and the intensity of the bands was calculated as the ratio of the indicated band to the GAPDH band. The primary antibodies included anti-Jagged2 (2210T, Cell Signaling Technology), anti-Collagen I (bs-10423R, Bioss), anti-alpha sma (bs-10196R, Bioss), anti-Notch1 (ab52627, Abcam), anti-NICD [Cleaved Notch1 (val1744) (D3B8)] (4147S, Cell Signaling Technology), anti-Hes1 (ab108937, Abcam), anti-Hes5 (ab194111, Abcam) and GAPDH (BSAP0063, Bioworld).

**Reference**

1. Chen Y, Wu Q, Lin J, Wei J. DARS-AS1 accelerates the proliferation of cervical cancer cells via miR-628-5p/JAG1 axis to activate Notch pathway. *Cancer Cell Int* 20:535, 2020.

2. Ning BF, Ding J, Yin C, Zhong W, Wu K, Zeng X, Yang W, Chen YX, Zhang JP, Zhang X, Wang HY, Xie WF. Hepatocyte nuclear factor 4 alpha suppresses the development of hepatocellular carcinoma. *Cancer Res* 70(19):7640-7651, 2010.

3. Wang J, Zhu CP, Hu PF, Qian H, Ning BF, Zhang Q, Chen F, Liu J, Shi B, Zhang X, Xie WF. FOXA2 suppresses the metastasis of hepatocellular carcinoma partially through matrix metalloproteinase-9 inhibition. *Carcinogenesis* 35:2576-83, 2014.

4. Ota M, Sasaki H. Mammalian Tead proteins regulate cell proliferation and contact inhibition as transcriptional mediators of Hippo signaling. *Development* 135:4059-4069, 2008.

5. Xu WP, Yi M, Li QQ, Zhou WP, Cong WM, Yang Y, Ning BF, Yin C, Huang ZW, Wang J, Qian H, Jiang CF, Chen YX, Xia CY, Wang HY, Zhang X, Xie WF. Perturbation of MicroRNA-370/Lin-28 homolog A/nuclear factor kappa B regulatory circuit contributes to the development of hepatocellular carcinoma. *Hepatology* 58:1977-1991, 2013.

6. Sasaki H, Hui C, Nakafuku M, Kondoh H. A binding site for Gli proteins is essential for HNF-3beta floor plate enhancer activity in transgenics and can respond to Shh in vitro. *Development* 124:1313-22, 1997.

7. Wrana JL, Attisano L, Cárcamo J, Zentella A, Doody J, Laiho M, Wang XF, Massagué J. TGF beta signals through a heteromeric protein kinase receptor complex. *Cell* 71:1003-14, 1992.

**Supplementary Figures**


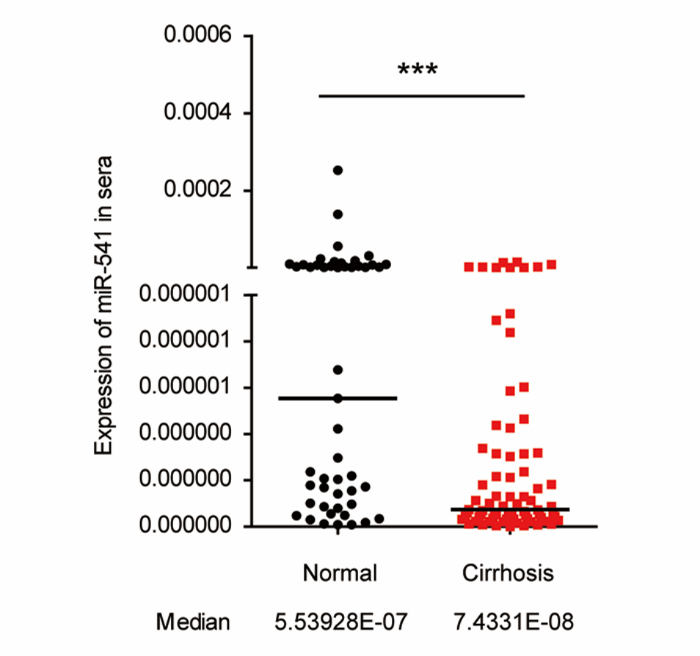


**Figure S1. The expression of miR-541 was downregulated in the serum of cirrhotic patients.**

Real-time PCR analysis of the expression of miR-541 in sera of healthy controls and patients with cirrhosis, with medians of 5.53928×10^−7^ and 7.4331×10^−8^, respectively; *p* < 0.0001, non-parametric Mann-Whitney test.


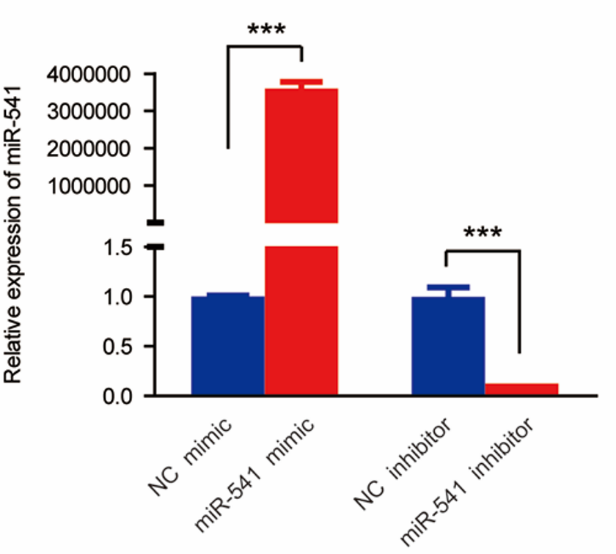


**Figure S2. Expression of miR-541 in LX-2 cells with miR-541 overexpression an inhibition.**

Real-time PCR analysis of the expression of miR-541 in LX-2 cells transfected with miR-541 mimic or miR-541 inhibitor and their corresponding negative control (NC). *** *p* < 0.001 by two-tailed Student’s t-test.


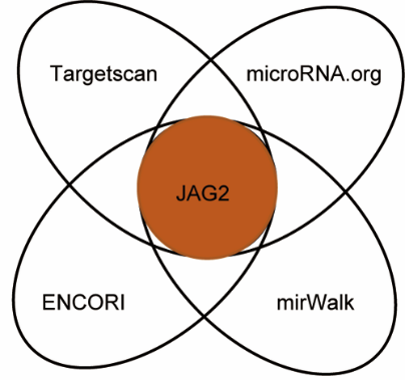


**Figure S3. All of the four miRNA prediction programs indicated JAG2 as a candidate target of miR-541.**


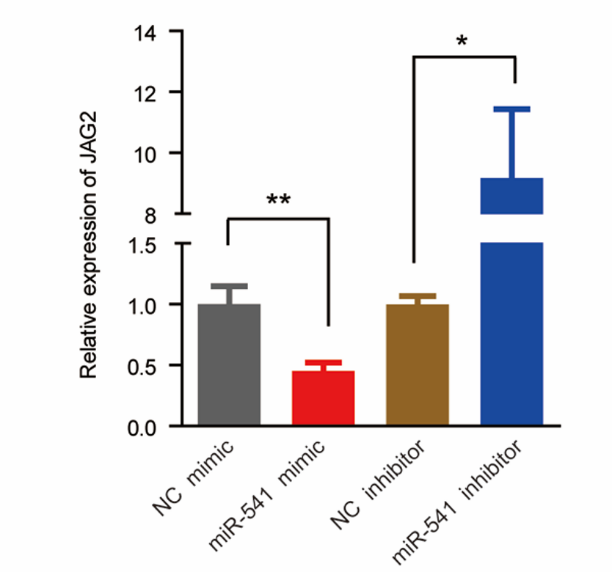


**Figure S4. The effect of miR-541 on the expression of JAG2.**

Real-time PCR analysis of JAG2 expression in LX-2 cells treated with TGF-β (5ng/ml) and transfected with the miR-541 mimic or miR-541 inhibitor and their corresponding negative control. * *p* < 0.05 and ** *p* < 0.01, two-tailed Student’s *t*-tests. Experiments were performed in triplicate and data are presented as means ± SD.


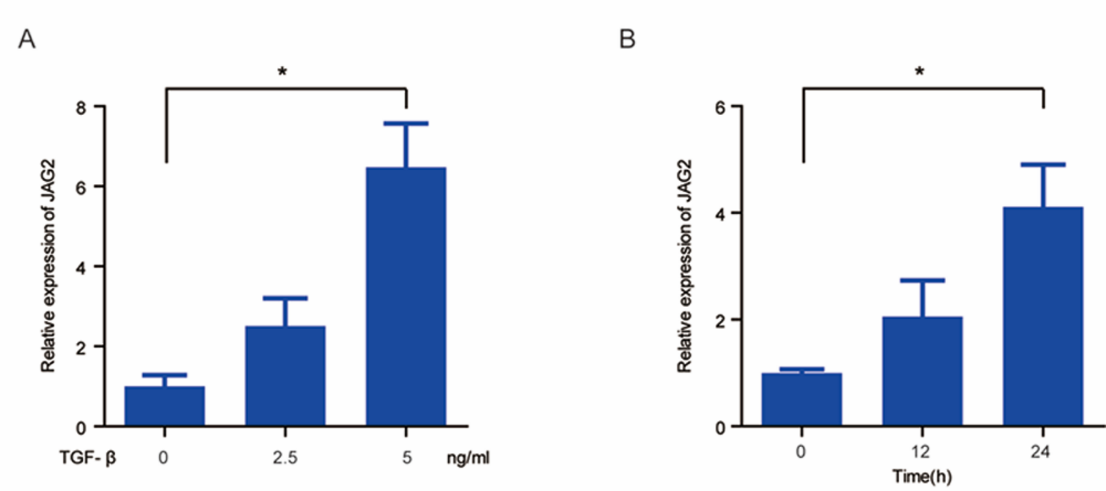


**Figure S5. The expression of JAG2 in the activated HSCs.**

(A) The expression of JAG2 was detected in LX-2 cells treated by 0, 2.5, 5 ng/ml TGF-β. (B) The expression of JAG2 was detected in LX-2 cells treated with TGF-β (5ng/ml) at 0h, 12h, 24 h. * *p* < 0.05 by two-tailed Student’s t-test. Experiments were performed in triplicate and data are presented as means ± SD.


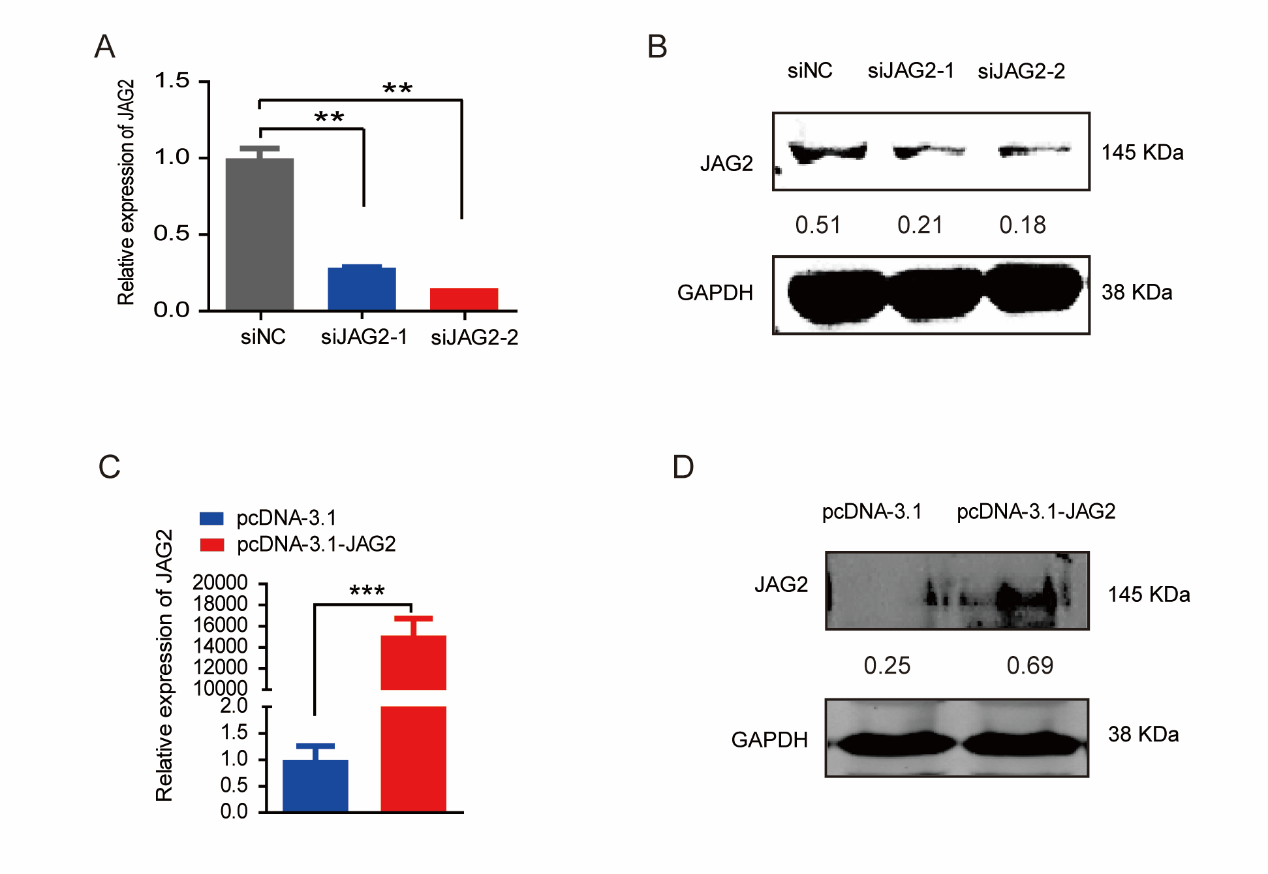


**Figure S6. Verification of the effectiveness of siRNAs targeting JAG2 and the plasmid overexpressing JAG2.**

(A and B) Expression of JAG2 mRNA (A) and protein (B) in activated LX-2 cells transfected with siJAG2 and negative control (siNC). (C and D) Real time PCR (C) and western blot (D) were used to investigate the expression of JAG2 in activated LX-2 cells transfected with overexpression plasmid and control plasmid. ** *p* < 0.01 and *** *p* < 0.001 by two-tailed Student’s t-test. Experiments were performed in triplicate and data are presented as means ± SD.


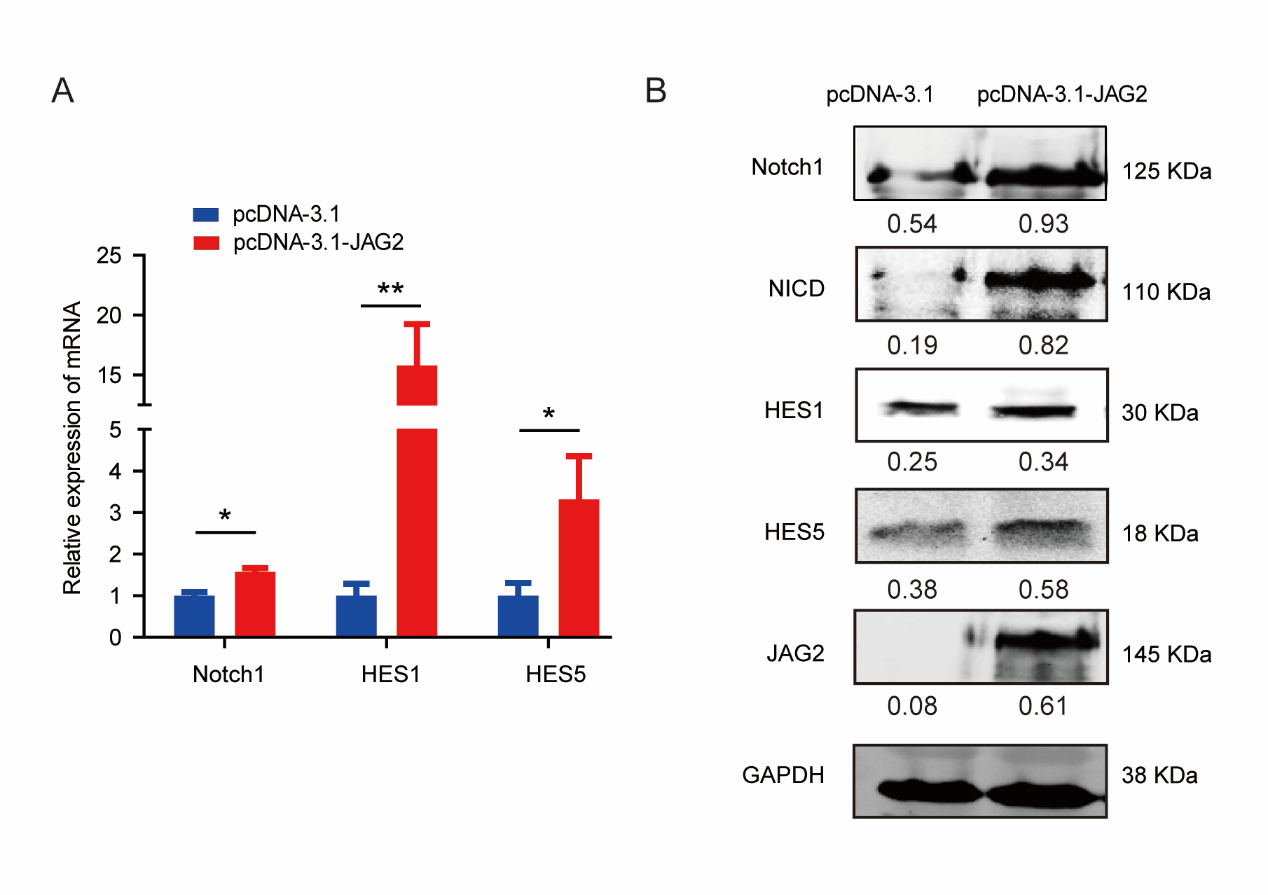


**Figure S7.** **Overexpression of JAG2 activated Notch signaling pathway.**

(A) Real-time PCR and (B) western blot analysis of the effects of JAG2 on the expression of key effectors of Notch signaling including Hes1, Hes5, Notch1. * *p* < 0.05 and ** *p* < 0.01 by two-tailed Student’s t-test. Experiments were performed in triplicate and data are presented as means ± SD.


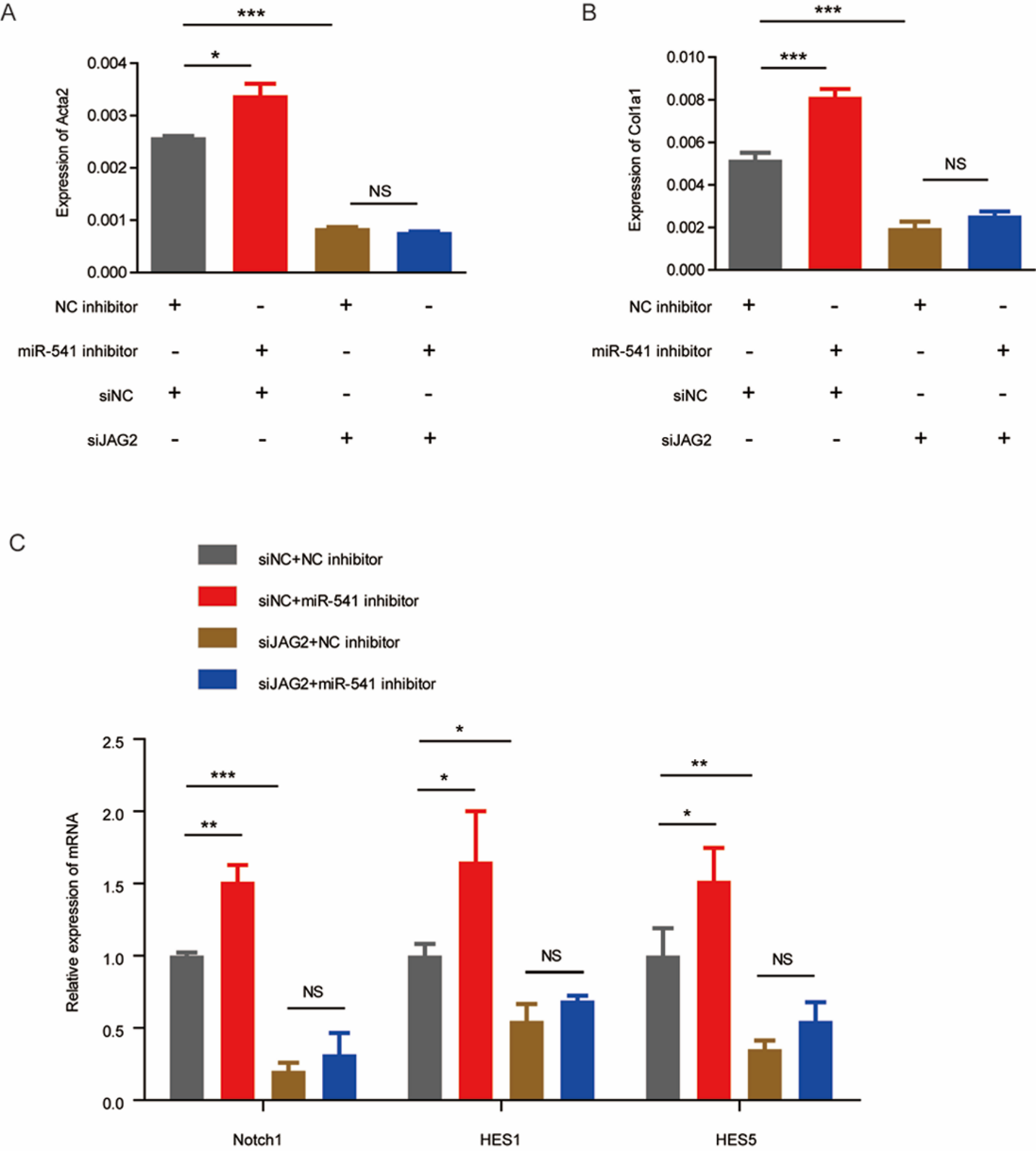


**Figure S8.** **siJAG2 partially reversed the effects of miR-541 inhibitor on the activation of HSCs and Notch signaling.**

LX-2 cells were co-transfected with siJAG2 or the negative control (siNC) along with the miR-541 inhibitor or negative control (NC). (A and B) The effect of the miR-541 inhibitor on Acta2 (A) and Col1a1 (B) mRNA was reversed by siJAG2. (C) The effect of the miR-541 inhibitor on the mRNA expression of Notch1, HES1 and HES5 of LX-2 cells was reversed by siJAG2. * *p* < 0.05, ** *p* < 0.01 and *** *p* < 0.001 by two-tailed Student’s t-test. NS, not significant. Experiments were performed in triplicate and data are presented as means ± SD.


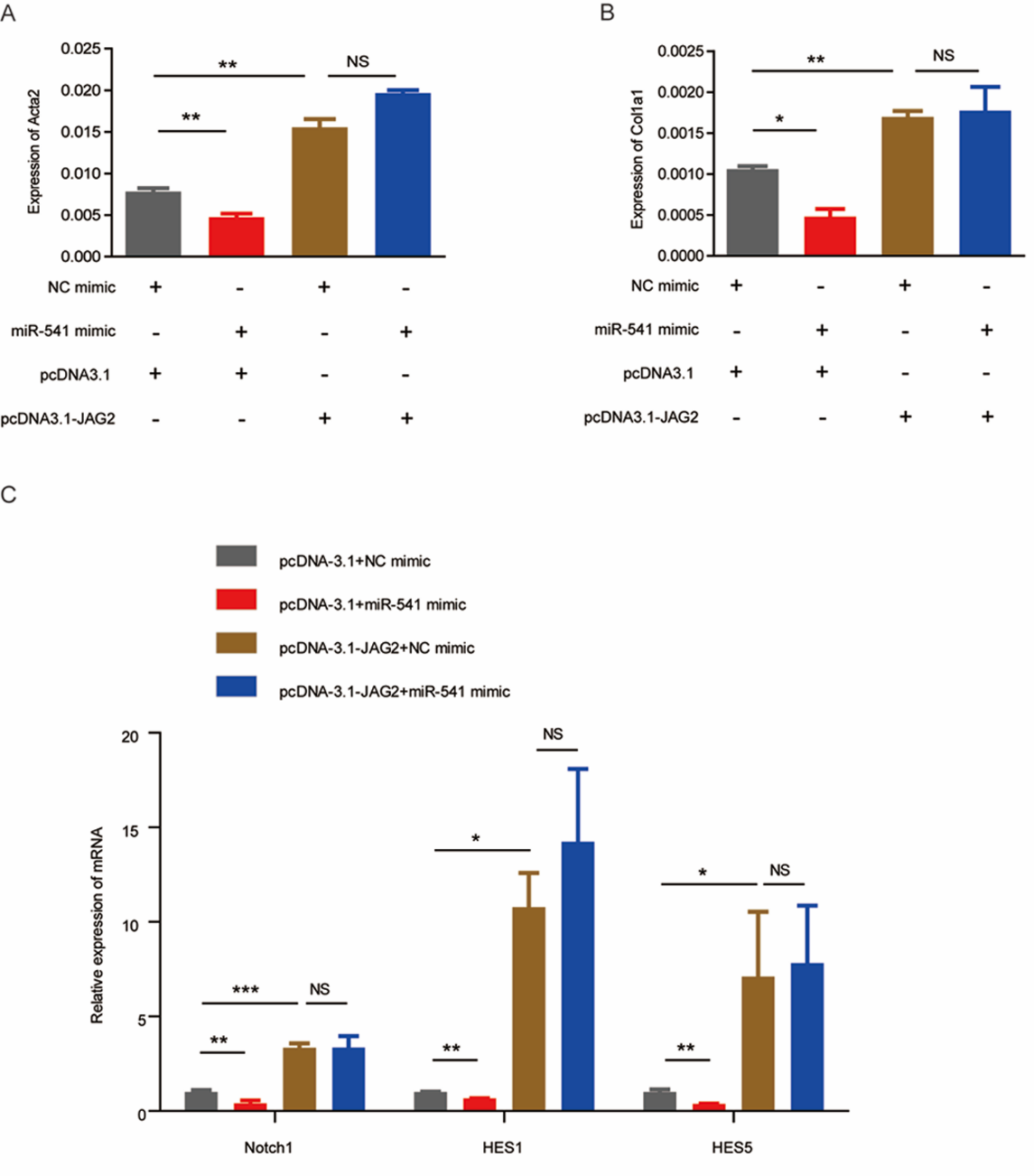


**Figure S9.** **Upregulation of JAG2 partially reversed the effects of miR-541 mimic on the activation of HSCs and Notch signaling.**

LX-2 cells were co-transfected with overexpression plasmid of JAG2 or the control plasmid along with the miR-541 mimic or negative control (NC). (A and B) The effect of the miR-541 mimic on Acta2 (A) and Col1a1 (B) mRNA was reversed by overexpression of JAG2. (C) The effect of the miR-541 mimic on the mRNA expression of Notch1, HES1 and HES5 of LX-2 cells was reversed by overexpression of JAG2. * *p* < 0.05, ** *p* < 0.01 and *** *p* < 0.001 by two-tailed Student’s t-test. NS, not significant. Experiments were performed in triplicate and data are presented as means ± SD.

**Supplementary Tables**

**Supplementary Table 1. Clinical characteristics of the patients in the serum cohort.**

|  | **Patients at enrollment** | **Patients with follow-up** |
| --- | --- | --- |
| **Characteristic** |  |  |
| N | 84 | 80 |
| Age at entry (years) | 57(35-79) | 57(35-79) |
| Male/Female | 45/39 | 41/39 |
| **Etiology of liver cirrhosis** |  |  |
| HBV | 46 | 42 |
| Alcoholic | 12 | 12 |
| PBC | 12 | 12 |
| Others | 14 | 14 |
| **Laboratory data at entry**  Platelet count (×10^9^  /L)  Albumin (g/L)  ALT (IU/L)  AST (IU/L)  ALP (IU/L)  GGT (IU/L)  Total bilirubin (mg/dl)  PT (s)  INR  miR-541(10E-4)  **Liver function**  Child-Pugh class，A/B/C | 97 (15-502)  33 (18-53)  36 (6-247)  55 (15-494)  141 (54-457)  83 (10-680)  50 (8-695)  16.6 (12.5-27.6)  1.37 (0.94-2.66)  1.47 (1.46E-6-111.01)  34/31/18 | 97 (15-502)  33 (18-53)  37 (6-247)  57 (15-494)  143 (54-457)  84 (10-680)  51 (8-695)  16.5 (12.5-27.6)  1.37 (0.94-2.66)  1.54 (1.46E-6-111.01)  33/30/16 |

Values are the medians with ranges in parentheses.

HBV, hepatitis B virus; PBC, primary biliary cirrhosis; ALT, alanine aminotransferase; AST,

aspartate aminotransferase; ALP, alkaline phosphatase; GGT, gamma-glutamyl transpeptidase; PT, prothrombin time; INR, international normalized ratio; AFP, α-fetoprotein.

**Supplementary Table 2. Sequences of primers used in Real-time PCR**

|  | Sequences (5’-3’) |
| --- | --- |
| Human β-actin forward | CATCCTGCGTCTGGACCT |
| Human β-actin reverse | GTACTTGCGCTCAGGAGGAG |
| Human JAG2 forward | AGGTGGAGACGGTTGTTACG |
| Human JAG2 reverse | TTGCACTGGTAGAGCACGTC |
| Human acta2 forward | GAAGGAATAGCCACGCTCAG |
| Human acta2 reverse  Human Col1a1 forward  Human Col1a1 reverse  Human Notch1 forward  Human Notch1 reverse  Human Hes1 forward  Human Hes1 reverse  Human Hes5 forward  Human Hes5 reverse | TTCAATGTCCCAGCCATGTA  CTCCTCGCTTTCCTTCCTCT  GTGCTAAAGGTGCCAATGGT  TGCAGAACAACAGGGAGGAG  CAGGTTGTACTCGTCCAGCA  TGAGCACAGACCCAAGTGTG  CCTCGGTATTAACGCCCTCG  CCGGTGGTGGAGAAGATG  GACAGCCATCTCCAGGATGT |

.

**Supplementary Table 3. Sequences of Oligonucleotides in this Study**

| Oligonucleotides | Sequences (5′-3′) |
| --- | --- |
| hsa-miR-541-3p mimic | UGGUGGGCACAGAAUCUGGACU |
| Negative control for miRNA mimic (NC mimic) | UUGUACUACACAAAAGUACUG |
| hsa-miR-541-3p inhibitor | AGUCCAGAUUCUGUGCCCACCA |
| Negative control for miRNA inhibitor (NC inhibitor) | CAGUACUUUUGUGUAGUACAA |
| siJAG2-1 sense | GCAAGGAAGCUGUGUGUAATT |
| siJAG2-1 antisense | UUACACACAGCUUCCUUGCTT |
| siJAG2-2 sense | GCAUCAACUGCCAUAUCAATT |
| siJAG2-2 antisense | UUGAUAUGGCAGUUGAUGCTT |
| Negative control for siRNA (siNC) sense | UUCUUCGAACGUGUCACGUTT |
| Negative control for siRNA (siNC) antisense | ACGUGACACGUUCGGAGAATT |
