# Supplementary material for: miR-541 is associated with the prognosis of liver cirrhosis and directly targets JAG2 to inhibit the activation of hepatic stellate cells
Source: BMC Gastroenterol. 2024 Feb 23;24:84. doi: 10.1186/s12876-024-03174-2 (PMC10893617; doi:10.1186/s12876-024-03174-2)

**Original blots for Western blot assay**

**Note:**

1. It should be noted that during the Western blot assay, we first cut out the corresponding membrane according to the molecular weight of the target protein and then incubate the membrane with the primary antibody. Therefore, our original image is not a full-length membrane.
2. To make it clearer, we display some images with relatively high contrast. Meanwhile, we include multiple exposure images in this Supplementary Information file.


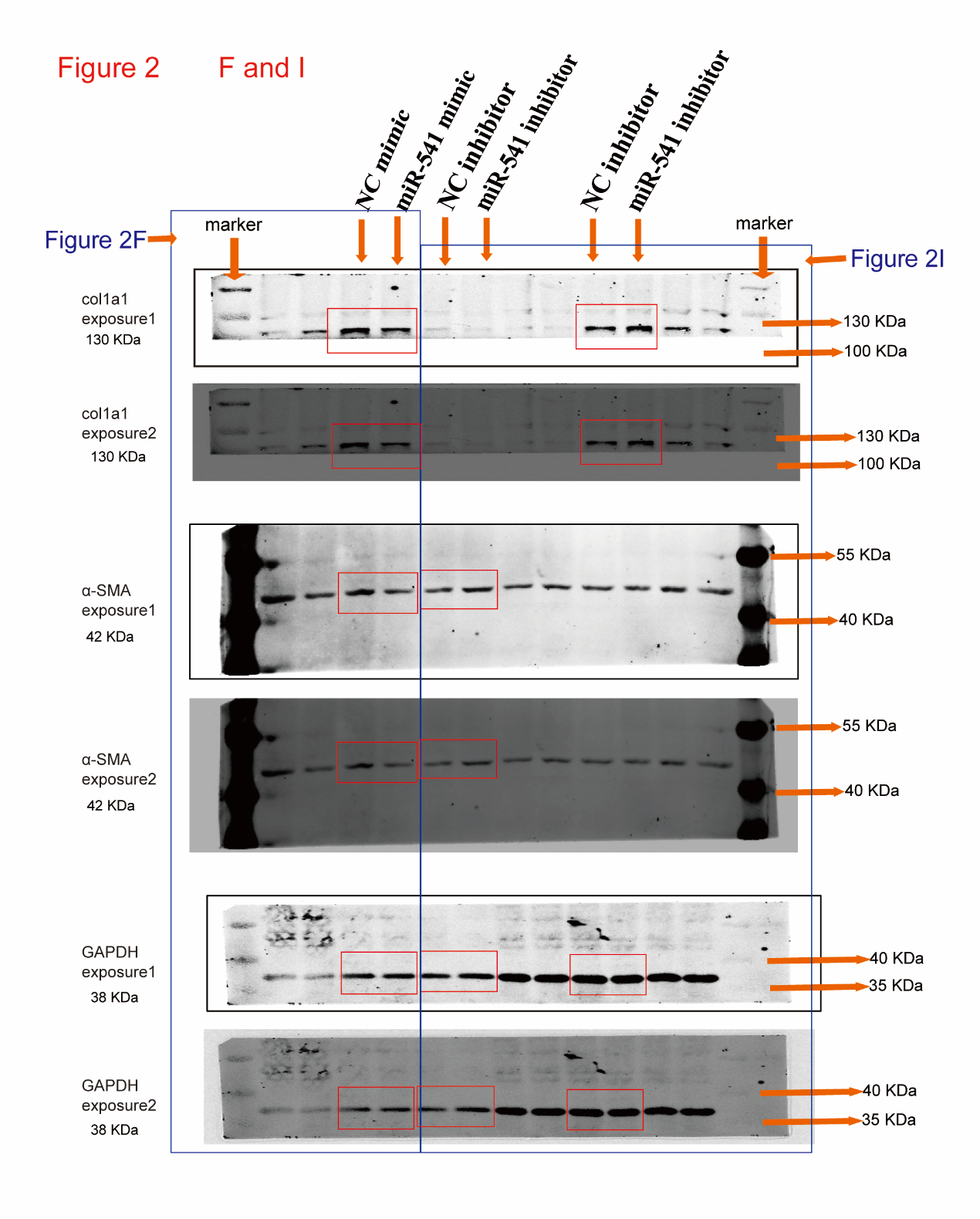

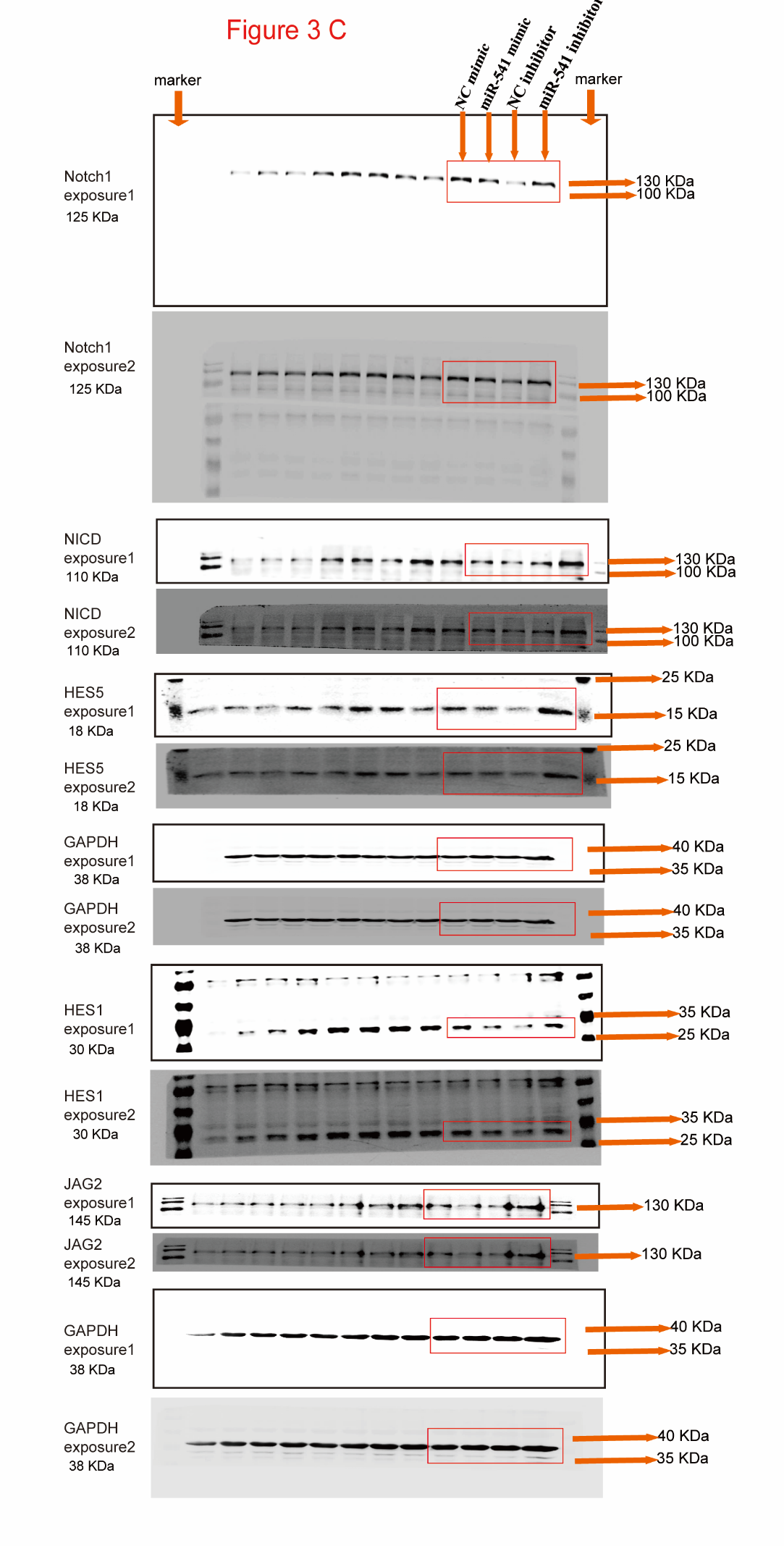

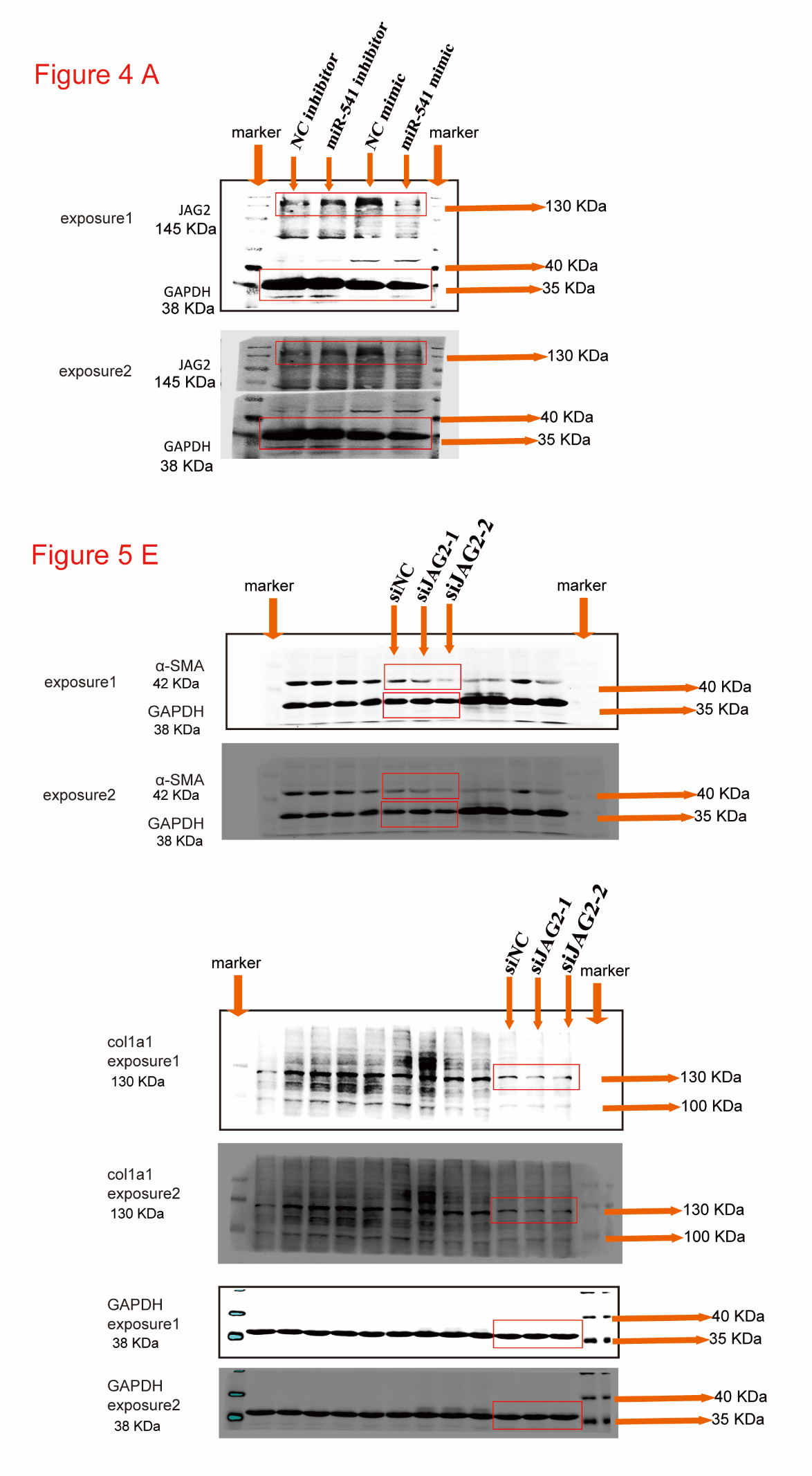

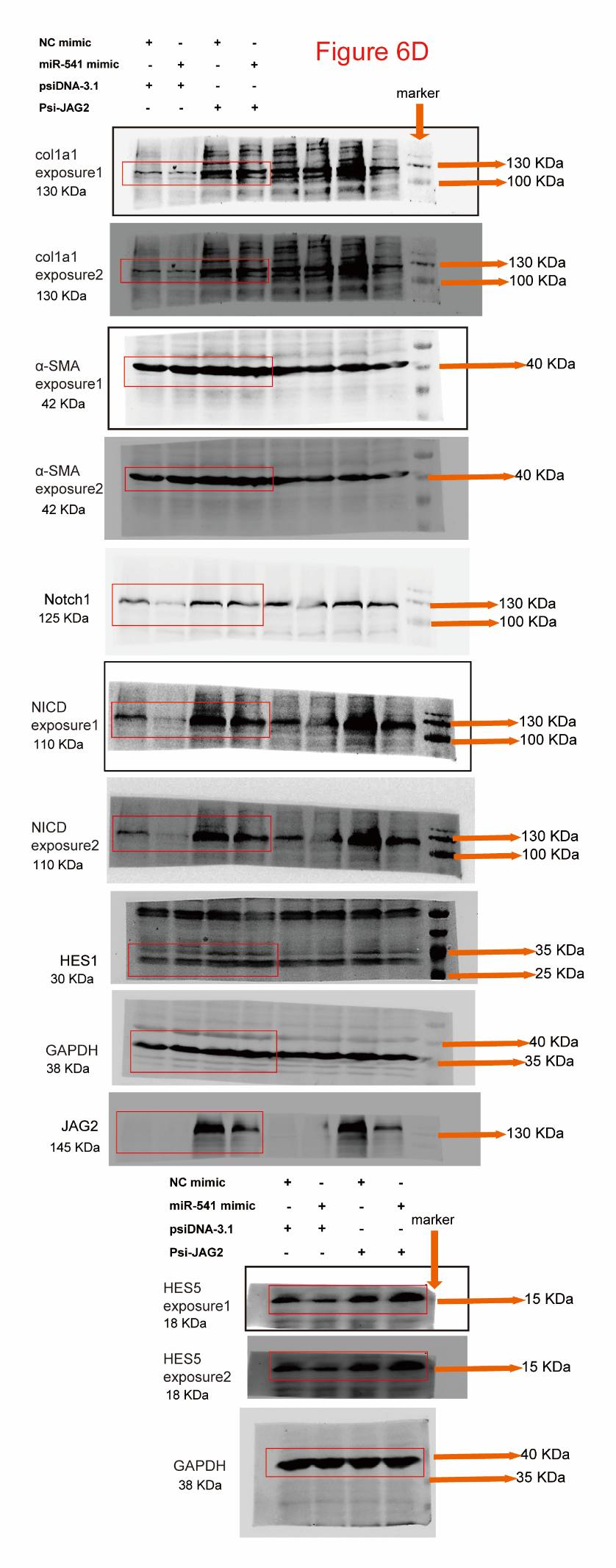

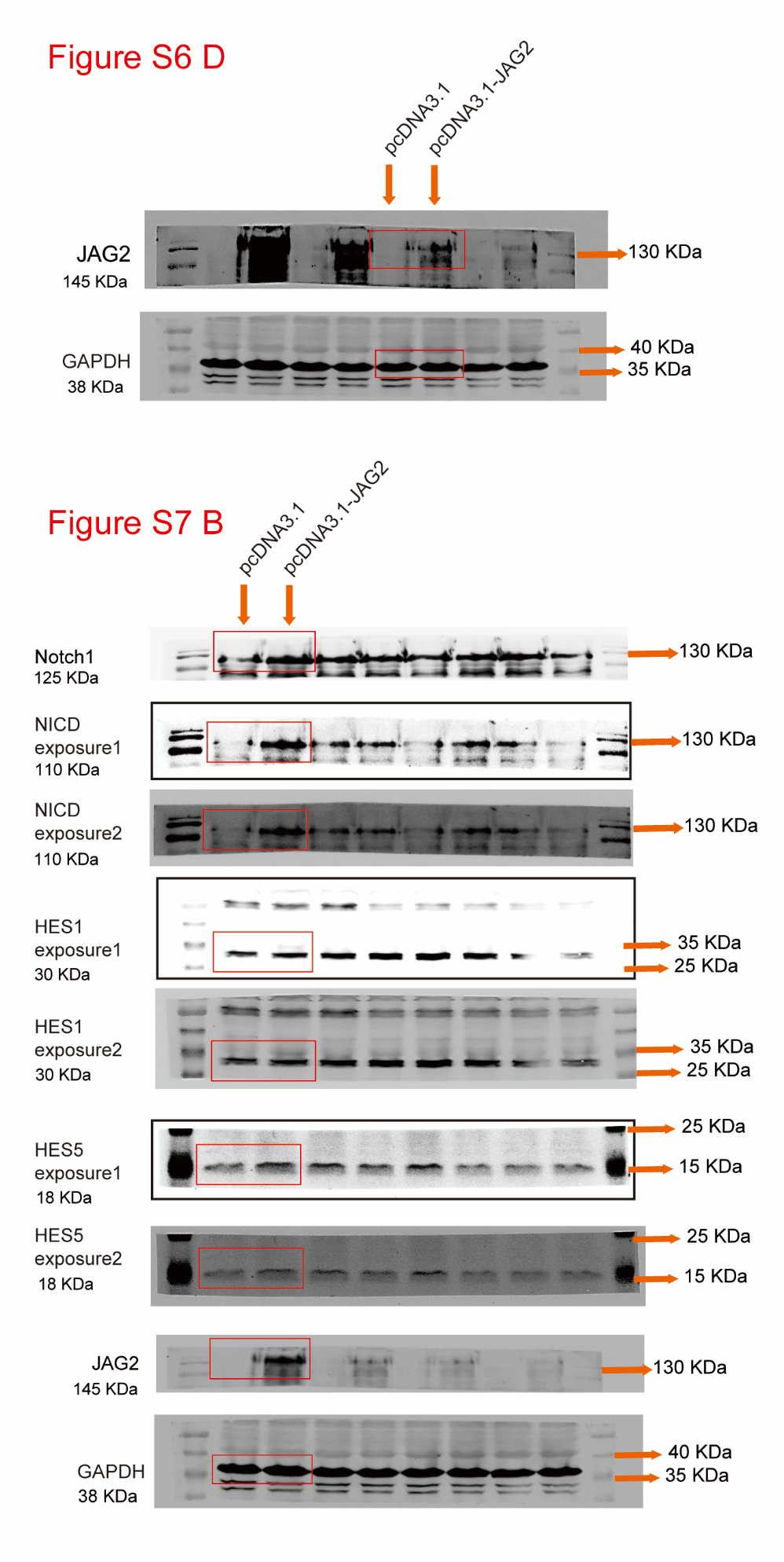

Supplement: Supplementary file 2 — Supplementary Material 2 [file 12876_2024_3174_MOESM2_ESM.docx]
